# Understanding intimate self-care among riverine women: qualitative research through the lens of the Sunrise Model

**DOI:** 10.1590/0034-7167-2023-0364

**Published:** 2024-07-19

**Authors:** Camila Evelyn de Sousa Brito, Bruna Larissa Fernandes Coelho, Talita Lima dos Santos, Thais Cristina Flexa Souza Marcelino, Lucas Geovane dos Santos Rodrigues, Mary Elizabeth de Santana, Antonio Jorge Silva Correa, Adriana de Sá Pinheiro

**Affiliations:** I Universidade da Amazônia. Belém, Pará, Brazil; II Universidade Federal do Pará. Belém, Pará, Brazil; III Universidade de São Paulo. Ribeirão Preto, São Paulo, Brazil

**Keywords:** Primary Health Care, Social Determinants of Health, Health Education, Transcultural Nursing, Women’s Health, Atención Primaria de Salud, Determinantes Sociales de la Salud, Educación en Salud, Enfermería Transcultural, Salud de la Mujer

## Abstract

**Objectives:**

to contribute to the change in understandings and knowledge of the popular system among riverine women about female intimate self-care before and after the application of an educational dynamic.

**Methods:**

a qualitative-participative study based on the Sunrise Model. Twenty women registered at a Basic Health Unit on the Combu island, state of Pará, Brazil, participated in the second half of 2022. Semi-structured interviews were conducted before and after the educational dynamic; followed by reflective inductive analysis.

**Results:**

these are pointed out: a female mechanism of generational education; the cultural act of bathing as synonymous with intimate self care and disease prevention; intimate care with medicinal herbs; lack of professional system approach to the topic; fear of using “muddy water”; and lack of financial resources to purchase specific products for genitourinary care.

**Final Considerations:**

companionship and social factors drive intimate self-care; however, riverine women experience taboos, ignorance, and poverty.

## RESUMEN

**Objetivos:**

contribuir para el cambio de comprensiones y saberes del sistema popular de mujeres ribereñas sobre el autocuidado íntimo femenino, antes y detrás la aplicación de una dinámica educativa.

**Métodos:**

estudio cualitativo-participativo apoyándose en el *Sunrise Model*. Participaron 20 mujeres, registradas en Unidad Básica de Salud de la isla de Combu, estado de Pará, Brasil, en el segundo semestre de 2022. Ocurrieron entrevistas semiestructuradas antes y detrás la dinámica educativa; y posterior análisis inductiva reflexiva.

**Resultados:**

señálese un mecanismo femenino de educación generacional; acto cultural del baño como sinónimo de autocuidado íntimo y prevención de enfermedades; aseos íntimos con hierbas medicinales; falta de abordaje del tema por el sistema profesional; temor del uso del “agua turbia”; y falta de recursos financieros para compra de productos específicos vueltos a cuidados genitourinarios.

**Consideraciones Finales:**

factores de compañerismo y sociales mueven el autocuidado íntimo, pero tabús, desconocimiento y pobreza han vividos por ribereñas.

## INTRODUCTION

Historically, traditional Brazilian populations living along riverbanks have been deprived of access to health services, adequate transportation, formal education, potable water, and basic sanitation, creating an unfavorable environment for disease control and prevention^(1)^. With the rubber crisis in Brazil at the end of the 19th century, the remaining workforce experienced a lack of public support policies, and workers dispersed along the riverbanks. This migration intensified on the islands of the municipality of Belém, where the riverine people established trade exchanges, managing natural resources and becoming a reference in the relationship between humans and nature^(2)^.

Their homes are built on stilts, with little or no access to electricity and running water, moving around in boats, canoes, and “rabetas”-small, agile boats with engines attached to the hull. The health risks they face due to economic disadvantage are emphasized, associated with the high costs of river transport considering the distance to more resourceful municipalities^(1)^.

In this context, the health care of riverine women, who, in addition to playing a central role in the sustenance of the home, need to take care of themselves and their family members, is highlighted^(3)^. In such a situation, nurses need to combine socio-anthropological reflection with their actions in the community, recognizing health problems and determinants, as well as strengthening the bond established in consultations and actions at health network points. It is believed that cultural training promotes or enhances these cultural competencies from the beginning of their education^(4)^.

Considering this scenario, female intimate hygiene is a form of disease prevention (vaginal discharge, vulvovaginitis, bacterial vaginosis, candidiasis, trichomoniasis, urinary tract infection, among others) and health promotion; therefore, implementing strategies to educate them about intimate self-care is necessary^(5)^. Given this, it is pertinent to address this issue based on the transcultural care of Madeleine Leininger’s theory (1925-2012), which amplifies the importance of the nurse’s practice, giving him more autonomy to act based on the complexity of the individual^(6)^.

Transcultural nursing is challenged to seek social justice through its care, considering themes of racism, diversity, equity, and health education^(7)^. From this perspective, the nurse must know the social determinants of health to size and adapt effective care, interventions, and guidance for the community. As an example of populations that require such conduct, there are the riverine people, who inhabit areas originating on the banks of rivers, lakes, and streams. A recent study in the state of Amazonas reinforces that such populations access health through small and medium-sized wooden boats; these residents frequently seek the hospital and have difficulties in scheduling appointments^(1)^.

This research aimed to work with women embedded in a traditional riverine population, considering their knowledge derived from what the academic system refers to as “common sense” or “popular knowledge”. For this, the conceptual support of the Sunrise Model from the Theory of Transcultural Care was used. Madeleine Leininger highlighted the cultural bases of caring in any group in promoting well-being, health, growth, and coping with disability or death, explaining structures, cultural congruence, intracultural variation, and some universalities^(8)^.

Her theory encompasses the worldview of users (the internal symbolic universe of each group that qualifies forms of care), dimensions of social structure, and professional and popular systems. Since the launch of the Theory in the early 1950s, nursing research has dealt with holistic comparative modes of care and healing. The theorist calls for transcultural nursing to produce more differential contributions^(8-9)^.

Given the above and with the empirical perception of the deficiency in intimate self-care among riverine women, it is questioned: How would knowledge be modified through applying a dynamic with riverine women from the Combu Islands, in the state of Pará, to encourage the performance of appropriate intimate self-care?

## OBJECTIVES

To contribute to the change in understandings and knowledge of the popular system among riverine women about female intimate self-care, before and after the application of an educational dynamic.

## METHODS

### Ethical Aspects

The investigation followed Resolution No. 510, from April 7, 2016, on the norms of research in human and social sciences, being approved by the Ethics Committees in Research of the proposing institutions. The participants signed the Informed Consent Form (ICF); and, to preserve confidentiality, sequential alphanumeric coding with the letter “P” for participants (P1, P2, P3 to P20) was used.

### Conceptual and Methodological Framework

The participative approach considers the experience and local determinants of the participants with the researcher’s mediation. Problematizing is key in the debate meetings, as the participative exercise nuances the participants’ interpretation of the phenomenon. A context or plot linked to the service and not just to the research project is pressing to allow the return of data and analysis of participation^(10)^.

More than a methodological strategy, the rounds, workshops, and actions that contribute to change/transformation, bursting shared experiences, cause mediation through testimony. They are a social positioning of the deponent and insertion of the investigation at a questioning level. Thus, the participative approach states that a status quo of omission interests a science of omission, whereas participative approaches promote care/preventive strategies with greater engagement^(10)^.

The Theory of Transcultural Care aims to give universal and differential explanations about health-illness-care. Its goal is to explain or provide culturally congruent, safe, and meaningful care. The Sunrise Model was chosen as conceptual support because it illustrates the theoretical assumptions and explains the foundations of each group’s intrinsic way of caring (values, beliefs, and practices of the person and families)^(11)^.

The worldview and caring dimensions constitute this model in seven factors: technological factors, religious and philosophical factors, companionship and social factors, cultural values and lifestyles, political and legal factors, economic factors, and educational factors. The actions of care based on the assessment of such factors are: Preservation of cultural care, Accommodation/negotiation, and Repatterning/restructuring^(11)^.

### Study Type

This is a descriptive research with a qualitative-participative approach. The domains “research team and reflexivity”, “study concept”, and “analysis and findings” were checked with the support of the Consolidated criteria for reporting qualitative research (COREQ), validated for Brazil^(12)^.

### Methodological Procedures

#### Study Setting

The geography of the Amazon is composed of sets of islands, among which 39 are in Belém, state of Pará (PA), Brazil. The Combu Island stands out, an environmental protection area bathed by the Guamá River. The setting was the Combu Basic Health Unit (UBS), which is part of the Guamá Administrative District (DAGUA), in the river channel known as “Igarapé do Combu,” whose geographical area is approximately 15 m^2(13)^. Activities occur from Monday to Friday, from 8 a.m. to 4 p.m., with individual care, medication dispensing, immunization, and collection of the Pap smear^(14)^.

#### Data Sources

Data collection took place from September to October 2022. Twenty women participated, all over 18 years old, sexually active, residing exclusively on Combu Island, and registered at the UBS.

It is worth mentioning that qualitative research should not be guided solely by quantity but also related to the dimension of the phenomenon and the quality of the actions^(15)^. Intentional sampling does not exempt the researcher from obtaining depth of perspectives; and, in participative procedures, generalizations are possible through theoretical-conceptual means and the set of relationships studied^(10)^.

That said, the five incursions of the researchers into the sample capture occurred as follows: in each incursion, four or five registered women were invited to answer the script before the dynamic; then, they participated in the educational strategy and afterward responded to the same script after the dynamic. A total of 25 women participated in the activity, but there was a loss of 5 women, as they did not have the time available to respond to the questions after the dynamic. Therefore, through this recruitment method, the study counted on the testimonies of 20 women, for participating in the collection stages and educational action.

#### Data Collection and Organization

Three nursing students conducted semi-structured interviews of nine questions, lasting up to 15 minutes each. They were aware of the research theme/scenario, and two researchers with experience in qualitative research trained them. It is noteworthy that it was the researchers’ first contact with the participants. The interviews and the educational action took place in a previously reserved room, and these stages involved only participants and researchers. In the first stage, there were six questions (pre-dynamic) before the board game, followed by a round of the game, and then interviews with three open questions in the post-dynamic stage. The responses were audio-recorded, converted to MP3 format, and subsequently transcribed into individual Microsoft Word files.

#### Stages, Resources, and Dynamics of Data Collection

The semi-structured modality follows a previously determined script, but it is adapted to the participant^(16)^. The questions asked before the dynamic were: 1) What do you understand by the expression female intimate self-care?; 2) Who taught you to have this type of care? Do you remember the age?; 3) What are the activities you do in your daily routine that are part of your intimate self-care?; 4) Have you sought professional help for more information? Which professionals?; 5) How do you perform female intimate self-care?; 6) What is inappropriate in performing female intimate self-care?. The questions after the dynamic were: 1) What are the activities you do in your daily routine that are part of your intimate self-care?; 2) After the game, how would you perform your intimate self-care?; 3) What is inappropriate in performing your intimate self-care? It is clarified that the term “inappropriate” was used to check the knowledge acquired after the dynamic, which could reveal a favorable effect of the methodology.

**Figure 1:**
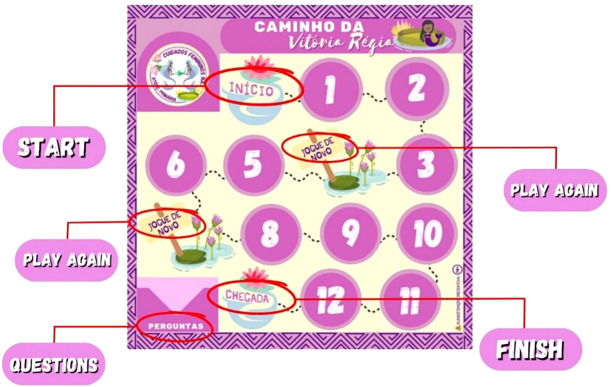
The Victoria Regia Path Magnetic Board, Belém, Pará, Brazil, 2023

The pedagogical resources used included: a metal board made of magnetic material (Figure 1), a plotted board composed of 13 spaces; 6 magnet pawns in different colors (green, yellow, lilac, blue, red, and pink) to identify the participants; and a space designated for the storage of question cards. There was also a large die (30 × 30 cm with six customized sides and images that refer to folkloric traits), 24 cards with “true or false” questions (about female intimate self-care, with the answers on the back), and a pamphlet with “Rules and Instructions”.

The game was developed for a minimum of two and a maximum of six participants, to be completed within 20 minutes. The design of the materials, developed on the Canva Pro platform, is based on board games. The cards are numbered and have images on the back (12 appropriate assertions and 12 inappropriate assertions), based on literature related to female intimate self-care. The chosen themes were: female genital anatomy, genital hygiene, types of discharge (and what causes them), use of condoms, sexually transmitted infections, and the Pap smear. The name “Victoria Regia Path” was chosen because the Victoria regia flower originates from the Amazon region, an aquatic plant with influence in the folklore of the North Region of Brazil, symbolizing the devotion of the indigenous Naiá to the moon goddess, Jacy^(17)^.

#### Data Analysis

In this study, software was not used: the analysis was manual with the validation of four coders, emerging from the set of testimonies. The inductive content reference of six stages and the type of reflective thematic analysis were used^(18-19)^. Thus, by the Sunrise Model and the participatory strategy, the data from the moment before and the moment after the dynamic were analyzed separately, to verify which the modifications in knowledge were. The researchers familiarized themselves with the transcriptions in search of patterns in the reading of each interview transcribed individually in Microsoft Word; subsequently, individual interviews were combined into a single Microsoft Word file (matrix); codes were generated based on the identification of “latent codes” (those beyond the semantic meaning given by the participants, providing an interpretation of the content) revealing the seven factors of Leininger with an inductive line of reasoning; themes were sought, in which the codes generated in the previous phase were grouped by the proximity of meanings and segregated into other Microsoft Word files; refinements and conferences of the generated themes took place with a re-reading of the files; there was the definition and naming of themes considering transculturality; and the final report was obtained. These steps occurred in the matrix file of the testimonies before and after the dynamic.

## RESULTS

Of the 20 women, the majority were single, with the predominant age range between 25 and 45 years, and an average of two children. They survived on a monthly income of 300 to 600 reais, i.e., 60 to 120 dollars (less than the current minimum wage, which is 1,302 reais [262 dollars]), with fishing and extractivism being the predominant professions, with an increase in income during the açaí (*Euterpe oleracea*) harvest season.

The data analysis culminated in the construction of Chart 1. Explaining the themes created in the light of Leininger’s scientific knowledge and the two-stage collection, the following care factors are identified^(8)^: Theme 1 - companionship and social factors, cultural values and lifestyles, educational factors, and technological factors; Theme 2 - religious and philosophical factors, political and legal factors, and economic factors. The latent codes related to preservation, accommodation, and repatterning were grouped into Theme 3, contemplating the decisions and actions of transcultural care from the Sunrise Model.

**Chart 1:** Themes Unveiled Before and After the Educational Strategy, Belém, Pará, Brazil, 2023

Data Production Moment	Themes
Pre-dynamic Testimonies	1) Association between culture and education in traditional ways of acquiring knowledge
	2) The impact of convictions and of favorable and unfavorable external resources for intimate care
Post-dynamic Testimonies	3) Verification of the need for adjustments, maintenance, and teaching of other congruent female care methods using the Victoria Regia Path

### Pre-dynamic: Association between Culture and Education in Traditional Ways of Acquiring Knowledge

Participants reported companionship and social factors when they remembered being educated by their mothers and grandmothers. These are teachings rooted in female family bonds.

*My mother taught me when I started menstruating.* (Summary P1, P2, P3, P4, P8)

*My mother, I think from the age of 5, already taught me to wash myself, just like I teach my daughter now.* (Summary P3, P6, P17)

*My grandmother, who has passed away today, taught me around 8 to 10 years old.* (Summary P7, P14, P16)

*It was my mother when I was a child. I think I was about 6 years old.* (Summary P9, P10, P11, P13, P15, P18, P20)

*My older sister taught me.* (Summary P12, P19)

Despite the teachings from the popular system, they feel the need for lectures and professional information, as they express indecision when choosing hygiene products.

*What makes intimate care easier is access to correct information, like lectures.* (Summary P1, P2, P10)

*About having many intimate soaps* […] *like, you know* […] *a person becomes indecisive about which one to use because, like, some can cause allergies and some that can cause itching, and this bothers me a lot, and the sanitary pads too.* (Summary P3, P12)

As educational factors, female intimate self-care related only to personal hygiene stood out, with bathing being the main means of execution. The care during menstruation alludes to what is considered incorrect, such as poor hygiene of the genital region.

*Cleaning wrongly* […] *many people don’t know the right way; sometimes, women don’t wash themselves and don’t know very well how they should do it.* (Summary P9 and P11)

*Wrong use of public bathrooms, because women sit down, but they shouldn’t; I think not washing after going to the bathroom is also wrong* […] *what if you get a disease.* (P18)

*I have to maintain good hygiene, right, take a bath, be careful during menstruation, wash my private parts, because I have to go to the doctor for a consultation, to get waxed.* (Summary P1, P2, P4, P5, P9, P13, P17, P20)

Some related female intimate self-care to the prevention of sexually transmitted infections and some diseases of the genital system, but did not identify or name which ones.

*I think it’s about preventing diseases* […]. *Being careful not to have sex without a condom and end up getting infected.* (Summary P8 and P10)

*I find it hard to find time to take care of myself because we are very busy there with things and also with fishing* […] *but we need to take some time to come here to the clinic.* (P7)

In accordance with cultural factors and lifestyle in agreement with educational factors, bathing is the main and sometimes only act of intimate care they perform:

*I do my intimate care in the shower* […]. *Like in the morning, when I get up, I do my hygiene: I use an intimate soap and then change clothes; later, I take a shower and wash again, taking several showers, but I don’t use intimate soap in all the showers* […] *I also change my sanitary pad every two hours when I’m menstruating.* (Summary P1, P3, P4, P5, P7, P8, P9, P12, P14, P15, P16, P17, P18, P19, P20)

*When I go to the bathroom to pee and poop, I wash and dry my private parts; and you also have to use soap that is specific for that care.* (P11)

Among the technological factors in seeking care and guidance, there is a reluctance of most to seek the professional system to discuss the topic.

*I always consult with the gynecologist. When it’s here at the clinic, I look for the nurse!* (Summary P1, P2, P9, P11, P19)

*I have never sought information about these things, neither with doctors nor with hospital staff.* (Summary P3, P4, P5, P6, P7, P8, P10, P12, P13, P14, P15, P16, P17, P18, P20)

### Pre-dynamic: The Impact of Convictions and Favorable and Unfavorable External Resources for Intimate Care

Three of the participants feel embarrassed to discuss the topic with male professionals; thus, issues such as menstruation and sexuality are taboos.

*I find it inappropriate. I don’t really like talking about these things, especially about intimate parts, with a male doctor* […]. *Embarrassment.* (Summary P1, P5, and P12)

Political and legal factors often related to economic factors are mentioned in the lack of adequate infrastructure in public places or household work as hindrances or facilitators of intimate care.

*What makes it easy is not having itching, not having an infection, washing my private parts well; when there is the material at home, sometimes we don’t have the information about what is right, going to the doctor, to the hospital. What makes it difficult is when there is no money; another thing that facilitates this care is having running water. When there wasn’t any, it was more difficult because the water wasn’t very clean. When there was no running water, it was hard; had to bathe in the river or go fetch water from the well.* (Summary P4, P8, P9, P10, P13, and P19)

*What makes it easy is that we have plenty of water here, and we can take a shower anytime; and what makes it difficult at my house is that I don’t have a bathroom inside the house.* (P6)

*I have to work, take care of the house, the children, and I don’t have much time for this. I work all day.* (Summary P15 and P17)

*What makes it easy is having a bidet* […] *inside the house, there is. And what makes it difficult is not having quality water: the risk of catching something with river water is higher.* (P11)

### Post-dynamic: Verification of the Need for Adjustments, Maintenance, and Teaching of Other Congruent Female Care Methods Using the Victoria Regia Path

After the proposed activity, women referred to the use of typical regional herbs to aid in body cleaning, reporting ease of access and benefits from their use.

*I take a shower, clean myself, I use chamomile* [Matricaria recutita]*, rosemary* [Salvia rosmarinus] […] *that’s what makes it easy, because it’s easy to get.* (P1)

*Using the herbs facilitates my care because I already have them in the forest; I plant mint* [Mentha spicata]*, rosemary, and toothache plant* [Acmella oleracea]. (P8)

*I use some herbs that are in the backyard; sometimes, I take a bath, you know, like rosemary, basil* [Ocimum basilicum]*, and fennel* [Pimpinella anisum]*. The neighbors also have them. What’s really bad was when there was no clean water.* (P13)

Even after the activity, participants believed female intimate self-care is exclusively about bathing, and one facilitator is having running water in homes.

*I wake up, then I take a shower, brush my teeth* […]. *When I go to the bathroom, I wash myself, take a shower, several times a day; I apply moisturizer, and maintain cleanliness, hygiene; at noon, I take another shower and, before going to bed, another shower. What makes it easy is that I like taking showers, right* […] *I pay for clean water.* (P3, P5, P6, P14, P19, P20)

*What makes it easy is having running water and having the financial means to buy good quality products, while what makes it difficult is the lack of basic sanitation that harms the whole community - before, we used to wash with river water, right.* (P9, P11)

*The shower, right* […] *body cleanliness, I take care of the clothes, as you said, I wash my panties, let them dry in the sun, change sanitary pads, don’t use public bathrooms, right* […]. *I do the women’s exams* […] *the preventive one.* (P7, P12, P15, P16)

*I wash myself, especially after sexual relations; I clean myself with a bidet every time I use the bathroom.* (P11)

The adjustment of proper female intimate self-care was related to the correct use of hygiene products and the management of techniques and materials for intimate and bodily care. This result was obtained after the application of the educational dynamic.

*What you said about intimate soap* […] *that it’s common for us to see intimate soap and think that we can be using it directly, and it turns out that no, that I had heard of pH, but I thought it was the bar soap that altered it. Now no, I found out that intimate soap also alters* […]. *I wouldn’t have imagined that about intimate soap.* (P1)

*Having clean water and the right products facilitates cleaning the vaginal region. I learned that I should not use intimate soap every day nor inside the vagina, only around it, that now I can use coconut oil during sexual relations, that I should not wear a sanitary pad for more than four hours nor a panty liner every day nor thong panties. The use of herbs in my hygiene facilitates, as does waxing, leaving the panties drying on the clothesline, and what makes it difficult is not having a bathroom, not having money to buy the materials.* (Synthesis P4, P6, P8, P10, P12, P13, P16, P17, P18, P20)

*Inappropriate is the reuse of dirty panties, wearing a sanitary pad for too long. Sometimes, a woman uses the sanitary pad until it overflows, the use of that internal sanitary pad that can cause an infection; you talked about having to pee after intercourse.* (P11)

*What makes it easy is having access to the health clinic, having places nearby to buy material. What makes it difficult is not having running water. Now I know that water has a lot of bacteria, and we can catch some disease.* (P14)

The repatterning of knowledge in the face of a lack of knowledge about female intimate self care was widely reported.

*What makes it easy is having guidance, like today: you guided us so well, something that we never thought about and did not know and today found out, like drying panties in the bathroom many people do* […] *I knew it couldn’t be done, but didn’t know why, now I know, that it’s because it can cause fungus and diseases. I learned about the right soaps, the sanitary pad, the preventive exam that needs to be done. The coconut oil that I learned I can use, not to dry inside the vagina.* (Synthesis P3, P7, P8, P9, P10, P13, P15, P16, P17, P19, P20)

*Inappropriate is what you taught* […] *we have to take a bath after sexual intercourse, to do the hygiene. Many people don’t know this! Look, it was also good to know that after a sexual act, it is important to pee, which will help in cleaning the intimate parts of the woman.* (P7)

## DISCUSSION

From a historical perspective, the concepts of Colonial Brazil and the Republic are fundamental to understanding Amazon and its people. Currently, national and international movements influence changes in the educational development of these populations, so that knowledge based exclusively on the culture of indigenous people is blended with various cultural traits^(20)^. The traditional family structure accommodated the man as the main provider; however, this scenario has changed in the West through women working outside the home. In the riverine context, women directly assist in the family’s economic product, leading the education of other women as a factor of companionship, cultural values, and lifestyles. They learned about their bodies from their mothers and grandmothers, as there is an implicit duty to transmit such care and teachings^(21)^.

This trait is very striking and shows diversity in the riverine culture, compared to the case of young Filipino women, mostly Catholics, living in the United States: they reported that learning alone or even with their mothers was hesitant, practical, and not very explanatory, possibly due to the “traditional” Asian culture^(22)^. A study in the Indian reality similarly found that young women have little openness to talk about menstruation with family and even with gynecologists, corroborating that when girls and adolescents are poorly guided or do not discuss the topic, they generally develop genitourinary disorders^(23)^.

Under the educational aspects of the Sunrise Model, it is inferred that the existing problems require implementing suitable microsocial policies. The Brazilian Ministry of Social Development (MDS) established the National Policy for the Sustainable Development of Peoples and Traditional Communities (PNPCT) through Decree 6.040, on February 7, 2017, via the National Commission for the Sustainable Development of Traditional Communities (CNPCT)^(24)^.

The way women interpret the materiality of care differs among themselves but comes from the same paradigmatic perspective, in which cultural and natural symbols integrate into action, in the cultural act of “bathing” defined by the river. The Unified Health System (SUS) aims to reduce vulnerability by valuing rural-urban traditional practices and knowledge^(11,25)^. In this study, disease prevention comes from a cosmology that does not know and does not name exactly what the academic-biomedical system has established: urinary tract infection, vaginitis, and sexually transmitted infection^(26)^.

Furthermore, three women in the sample mentioned feeling embarrassed to talk with male professionals. There are consequences of rural patriarchal upbringing: the shame of their genitals and, more deeply, the violation of intimacy. Similarly, the patriarchal factor persists in Asian cultures; and whether the female gender has (or does not have) confidentiality is a fundamental factor in seeking a professional^(22)^. Shame can be combated in women’s groups, making it easier for them to undergo invasive procedures like Pap smear screening; and encouragement can be provided with actions and conversations in circles to demystify^(27)^.

The verbatim reports of the second theme expressed the aspiration for clean water and the fact that muddy water is harmful. This need finds support in Article 2 of the PNPCT, which provides for the main objective of promoting sustainable development, with an emphasis on guaranteeing rights. The implementation of the policy prompted the MDS to apply measures in response to the main problems of traditional communities, such as supporting projects that help structure family production, commercialization, and expanding access to water^(24)^. Traveling by rivers and the subsistence activities make the available time an obstacle in seeking the formal system^(1)^. The social participation of these women establishes them as a specific category in the fight against physical and symbolic exclusion, for being connected to the river. In Pará, this social participation has been strengthened due to the National Program to Support Family Agriculture (PRONAF), the Paraense Movement of Rural Education, and the Paraense Forum of Rural Education.

Economic factors are impediments to purchasing products, relegating women to embarrassment and “menstrual poverty.” The term refers to millions of menstruating individuals who, because of their socioeconomic profile, cannot afford hygiene products, lack potable water, or even sanitation that enables dignified intimate hygiene, making this natural process burdensome and causing, among other things, absence from work or school during the cycle^(28-29)^. One way found in popular knowledge is herbal baths, originating from indigenous tradition, favoring a natural therapy based on territoriality, religiosity, and healing rituals, which composes the riverine cosmology^(25)^. Intimate hygiene with herbs is rooted in a way of producing generational and millennial health; and, even with pharmacological advances, it is a commonplace use^(30)^.

This is similar to a study conducted in Ghana, an African country, which showed that women have positive perceptions of medicinal herbs based on indigenous knowledge, neighbors, family, and their autonomy. The use of herbs is due to cost-effectiveness and availability related to the following plants: chamomile (*Matricaria recutita*), rosemary (*Salvia rosmarinus*), mint (*Mentha spicata*), toothache plant (*Acmella oleracea*), basil (*Ocimum basilicum*), and fennel (*Pimpinella anisum*)^(31)^. At this point, from the perspective of the Sunrise Model, there is a confluence between economic factors and cultural values and lifestyles.

Another research^(32)^ advocates the use of medicinal herbs, emphasizing that this knowledge is considerably rooted in the culture and that, even having access to modern medicines, the choice to use herbs is the result of a personal choice. Thus, it is evident that the herbs chamomile, mint, fennel, rosemary, and basil, mentioned by the participants, are present in the articles cited earlier, indicating the cultural proximity between these contexts when it comes to disease treatment.

Similarly, comparing the results with the application of the Sunrise Model in Spanish villages, the theme “hygiene measures” was dominant, revealing that women use animal, plant, and mineral resources for body cleaning; and the theme “companionship”, showing how the community has the potential to embrace such caring practices. Finally, it is believed that the data reinforce the need for nursing actions aimed at generational female intimate self-care, not always visualized by the academic system, highlighting, in the popular system, older women as transmitters of this knowledge.

### Study limitations

The interviews were conditioned by the wait for consultations and the dynamics of care at the UBS, therefore the data produced in the context of the activity were significantly affected by this. Possibly, more available time for testimonies and the application of the dynamic in a different scenario would favor other thematic immersions.

### Contributions to the Field

Transcultural theory contributes to the identification of nursing problems, turning cultural care into a guiding principle of the Nursing Process^(33-34)^. By applying the Victoria Regia Path game, the gaps in formal knowledge about female intimate self-care became explicit. The figure of the nurse as the main mediator in the sharing of information within Primary Health Care (PHC) is highlighted, and the research helps in identifying the real needs, based on local limitations. It was understood that in health practices, the congruence of cultural care and the fulfillment of the principles of otherness are necessary, knowing that PHC should preserve most understandings. Health education is an important arbitration for the awareness of these people of the water and the forest, who do not ignore the need for teachings from the technical-scientific system and attest to the relevance of popular knowledge received from their peers, even knowing that they are not sufficient in terms of female intimate self-care.

## FINAL CONSIDERATIONS

The knowledge of riverine women about female intimate self-care has been modified; however, it was not intended to exclude their experiences and customs, which, upon deeper analysis, need to be recognized as cultural heritage to be preserved, endorsing the SUS public policies in the area of multiculturalism. Noteworthy in these knowledges is the vocalization of how participative education facilitated the understanding of information that is, according to companionship and social factors, only passed on by mothers, sisters, aunts, and grandmothers at some point in childhood puberty, with mothers being the primary transmitters of this generational and almost instinctive knowledge about the body. The Victoria Regia Path highlighted in a moment-space the “academic-professional system” together with the “popular-users system,” a form of health production.

Economic factors were strongly emphasized by participants, citing the lack of access to running water as one of the most relevant at the time of practicing their female intimate self-care. Therefore, during the performance of their hygiene as a repatterning of cultural care within the realm of knowledge, the presence of a certain fear of using the “muddy water” from the river for cleaning the vulva was reported, as it is untreated and contaminated. Thus, they reveal that they are aware it is not suitable because it may contain microorganisms that favor infections.

According to Madeleine Leininger’s Sunrise Model, the participants were able to receive new knowledge and will certainly apply and disseminate in their social-generational environment the appropriate forms of female intimate self-care, still in synergy with the nature surrounding the Combu Islands.

## Supplementary Material

0034-7167-reben-77-02-e20230364-Suppl01

0034-7167-reben-77-02-e20230364-Suppl02

0034-7167-reben-77-02-e20230364-Suppl03

0034-7167-reben-77-02-e20230364-Suppl04

0034-7167-reben-77-02-e20230364-Suppl05

0034-7167-reben-77-02-e20230364-Suppl06

0034-7167-reben-77-02-e20230364-Suppl07

0034-7167-reben-77-02-e20230364-Suppl08

0034-7167-reben-77-02-e20230364-Suppl09

0034-7167-reben-77-02-e20230364-Suppl10

0034-7167-reben-77-02-e20230364-Suppl11

0034-7167-reben-77-02-e20230364-Suppl12

0034-7167-reben-77-02-e20230364-Suppl13

0034-7167-reben-77-02-e20230364-Suppl14

0034-7167-reben-77-02-e20230364-Suppl15

0034-7167-reben-77-02-e20230364-Suppl16

0034-7167-reben-77-02-e20230364-Suppl17

0034-7167-reben-77-02-e20230364-Suppl18

0034-7167-reben-77-02-e20230364-Suppl19

0034-7167-reben-77-02-e20230364-Suppl20

## Data Availability

https://doi.org/10.48331/scielodata.8D4CF7
