# Supplementary material for: Understanding intimate self-care among riverine women: qualitative research through the lens of the Sunrise Model
Source: Rev Bras Enferm. 2024 Jul 19;77(2):e20230364. doi: 10.1590/0034-7167-2023-0364 (PMC11259441; doi:10.1590/0034-7167-2023-0364)
Supplement: 0034-7167-reben-77-02-e20230364-Suppl01 [file 0034-7167-reben-77-02-e20230364-Suppl01.pdf]

TRANSCRIÇÃO DE ENTREVISTA

PRIMEIRA ENTREVISTA - GRAVAÇÃO: **P1**

1. Idade: 43 anos
2. Estado Civil: casada
3. Filhos: sim
3.1 Se sim quantos: 2
4. Escolaridade: ens. Médio completo
5. Profissão: doméstica
6. Qual sua renda mensal (quantos salários-mínimos): 2 s. mínimo
7. Quantas pessoas moram na sua casa: 4

ENTREVISTA

O que você compreende quando escuta a expressão "cuidados íntimos"?

"Cuidados íntimos são... acho que os cuidados com nossas partes íntimas né, fazer o tratamento direito, a lavagem... acho que isso" – P1

Quem lhe ensinou a ter esse tipo de cuidado?

"Minha mãe me ensinou... e decorrência de ir ao médico né, o ginecologista... ah explicar essa situação de cuidados íntimos" – P1

A senhora lembra idade que começou pensar em cuidados íntimos?

"Não... hum não lembro" – P1

Quais são as coisas que você faz no dia a dia que fazem parte do seu cuidado íntimo?

"Olha o banho né com asseio e uso aqueles sabonetes íntimos" – P1

Já buscou ajuda profissional para ter mais informações sobre isso? Quais profissionais?

"o ginecologista" – P1

O que facilita ou dificulta a execução destes cuidados íntimos na sua opinião?

"O que facilita... acho que... o que facilita assim é a questão da gente ter consciência de que precisa ter esses cuidados, que nem quando a gente a gente já teve cuidados com certos incômodos, a gente ter as informações corretas" – P1

O que é inadequado na realização dos cuidados íntimos?

"Inadequado (pausa) o que eu acho inadequado, que eu não gosto muito assim... é de conversar sobre essas coisas principalmente da parte intima com homem médico...eu não gosto (riso)" – P1

SEGUNDA ENTREVISTA - GRAVAÇÃO: **P1**

Quais são as coisas que você faz no dia a dia que fazem parte do seu cuidado íntimo?

"Eu tomo banho.... ai eu tomo banho, ai me asseio, faço aquela ervas né medicinais pra fazer aqueles banhos pra fazer aqueles asseios..." – P1

O que facilita ou dificulta a execução destes cuidados íntimos na sua opinião?

"O que facilita tipo assim... é que as ervas a gente tem, é fácil da gente conseguir... acho que a dificuldade a gente não tem nenhuma" – P1

O que é inadequado na realização dos cuidados íntimos?

"Ah.. por exemplo o que vocês falaram do sabonete intimo né... que é comum a gente ver sabonete intimo e pensa que a gente pode ta utilizando direto né, e acaba não, que já tinha ouvido falar do ph, mas eu pensei que era o sabonete em barra que... alterava, agora não, eu fiquei sabendo que o intimo também altera... é sempre bom a gente ta sabendo mais, por exemplo eu não imaginaria isso do sabonete íntimo" – P1
