# Supplementary material for: Understanding intimate self-care among riverine women: qualitative research through the lens of the Sunrise Model
Source: Rev Bras Enferm. 2024 Jul 19;77(2):e20230364. doi: 10.1590/0034-7167-2023-0364 (PMC11259441; doi:10.1590/0034-7167-2023-0364)
Supplement: 0034-7167-reben-77-02-e20230364-Suppl02 [file 0034-7167-reben-77-02-e20230364-Suppl02.pdf]

TRANSCRIÇÃO DE ENTREVISTA

ENTREVISTA – PÓS DINÂMICA. GRAVAÇÃO: **P2**

1. Idade: 34 anos
2. Estado Civil: casada
3. Filhos: sim
3.1 Se sim quantos: 01
4. Escolaridade: Fundamental Completo
5. Profissão: Pescadora
6. Qual sua renda mensal (quantos salários-mínimos): 01 salário mínimo
7. Quantas pessoas moram na sua casa: 02 pessoas

ENTREVISTA

O que você compreende quando escuta a expressão "cuidados íntimos"?

"lavar as partes íntimas" – P2

Quem lhe ensinou a ter esse tipo de cuidado?

"indo ao médico" – P2

A senhora lembra idade que começou pensar em cuidados íntimos?

"desde criança" – P2

Quais são as coisas que você faz no dia a dia que fazem parte do seu cuidado íntimo?

"asseio, depilação, banho" – P2

Já buscou ajuda profissional para ter mais informações sobre isso? Quais profissionais?

"sim, do ginecologista" – P2

O que facilita ou dificulta a execução destes cuidados íntimos na sua opinião?

"facilita: palestras, informações. Dificulta: falta de acesso" – P2

O que é inadequado na realização dos cuidados íntimos?

"falta de algum conselho de como fazer direito" – P2

ENTREVISTA – PÓS DINÂMICA. GRAVAÇÃO: **P2**

Quais são as coisas que você faz no dia a dia que fazem parte do seu cuidado íntimo?

"troca de absorvente, limpeza após a relação, alimentação...– P2

O que facilita ou dificulta a execução destes cuidados íntimos na sua opinião?

"falta de informação" – P2

O que é inadequado na realização dos cuidados íntimos?

"não sei" – P2
