# Supplementary material for: Understanding intimate self-care among riverine women: qualitative research through the lens of the Sunrise Model
Source: Rev Bras Enferm. 2024 Jul 19;77(2):e20230364. doi: 10.1590/0034-7167-2023-0364 (PMC11259441; doi:10.1590/0034-7167-2023-0364)
Supplement: 0034-7167-reben-77-02-e20230364-Suppl03 [file 0034-7167-reben-77-02-e20230364-Suppl03.pdf]

TRANSCRIÇÃO DE ENTREVISTA

PRIMEIRA ENTREVISTA - GRAVAÇÃO: **P3**

1. Idade: 27 anos
2. Estado Civil: união estável
3. Filhos: sim
3.1 Se sim quantos: 2
4. Escolaridade: ens. Médico completo
5. Profissão: pescaria
6. Qual sua renda mensal (quantos salários-mínimos): 1 s. mínimo
7. Quantas pessoas moram na sua casa: 3

ENTREVISTA

O que você compreende quando escuta a expressão "cuidados íntimos"?

"Muita coisa.... que a gente tem que se cuidar né" – P3

Mas em relação a que... o que a senhora lembra quando eu falo "cuidados íntimos"?

"Ter mais atenção com o cuidado com as partes... também eu tenho uma filha né, então tenho que ter atenção sobre isso" – P3

Quem lhe ensinou a ter esse tipo de cuidado?

"Minha mãe... quando eu comecei a menstruar... ela me falou como era tudinho... explicou né.. eu tinha 11 anos" – P3

Quais são as coisas que você faz no dia a dia que fazem parte do seu cuidado íntimo?

"Na hora do banho... tomar banho e (pausa) sabe fazer a limpeza direito" – P3

Já buscou ajuda profissional para ter mais informações sobre isso? Quais profissionais?

"hum... não" – P3

O que facilita ou dificulta a execução destes cuidados íntimos na sua opinião?

"Sobre ter muitos sabonetes íntimos assim né... a pessoa fica indecisa qual usar, porque tipo tem uns que pode dar alergia tem uns que pode fazer coçar e isso me incomoda bastante sobre isso, ahh os absorventes também" – P3

O que é inadequado na realização dos cuidados íntimos?

"Usar aquela buchinha de banho" – P3

SEGUNDA ENTREVISTA - GRAVAÇÃO: **P3**

Quais são as coisas que você faz no dia a dia que fazem parte do seu cuidado íntimo?

“Limpeza né... ter o maior cuidado com a limpeza... higiene” – P3

O que facilita ou dificulta a execução destes cuidados íntimos na sua opinião?

“O que facilita saber usar e ter os sabonetes e absorventes... esses materiais e o que dificulta é não saber qual usar” – P3

O que é inadequado na realização dos cuidados íntimos?

“Uso da ducha e do absorvente íntimo de forma inadequada que vocês disseram né, o tempo que pode usar né... não fazer o preventivo é errado... porque eu sei que as mulheres tem vergonha de fazer” – P3
