# Supplementary material for: Understanding intimate self-care among riverine women: qualitative research through the lens of the Sunrise Model
Source: Rev Bras Enferm. 2024 Jul 19;77(2):e20230364. doi: 10.1590/0034-7167-2023-0364 (PMC11259441; doi:10.1590/0034-7167-2023-0364)
Supplement: 0034-7167-reben-77-02-e20230364-Suppl04 [file 0034-7167-reben-77-02-e20230364-Suppl04.pdf]

TRANSCRIÇÃO DE ENTREVISTA

ENTREVISTA – PÓS DINÂMICA. GRAVAÇÃO: **P4**

1. Idade: 43 anos
2. Estado Civil: solteira
3. Filhos: sim
3.1 Se sim quantos: 02
4. Escolaridade: Ensino Médio Incompleto
5. Profissão: Pescadora
6. Qual sua renda mensal (quantos salários-mínimos): menos de salário mínimo
7. Quantas pessoas moram na sua casa: 01 pessoa

ENTREVISTA

O que você compreende quando escuta a expressão "cuidados íntimos"?

"eu compreendo que eu tenho que fazer uma boa higiene né, a lavagem, tenho que ir ao médico, me consultar, fazer depilação..." – P4

Quem lhe ensinou a ter esse tipo de cuidado?

"minha mãe" – P4

A senhora lembra idade que começou pensar em cuidados íntimos?

"quando eu comecei a menstruar (13 anos de idade)" – P4

Quais são as coisas que você faz no dia a dia que fazem parte do seu cuidado íntimo?

"tipo de manhã quando eu levanto eu faço a minha higiene: passo um sabonete íntimo e depois troco de roupa, amis tarde tomo um banho e torno a lavar, tomando vários banhos, mas não uso sabonete íntimo em todos os banhos... também troco o meu absorvente de duas em duas horas quando estou menstruada." – P4

Já buscou ajuda profissional para ter mais informações sobre isso? Quais profissionais?

"não" – P4

O que facilita ou dificulta a execução destes cuidados íntimos na sua opinião?

"dificulta: a água, que não é uma água limpa" – P4

O que é inadequado na realização dos cuidados íntimos?

"acho que a questão da água" – P4

ENTREVISTA – PÓS DINÂMICA. GRAVAÇÃO: **P4**

Quais são as coisas que você faz no dia a dia que fazem parte do seu cuidado íntimo?

"lavava muito com sabão íntimo, lavar também com água a as partes íntimas, a depilação...". – P4

O que facilita ou dificulta a execução destes cuidados íntimos na sua opinião?

"estar sozinha em casa facilita, mas dificulta a questão de a água não ser ideal" – P4

O que é inadequado na realização dos cuidados íntimos?

"o excesso do sabonete íntimo e a água" – P4
