# Supplementary material for: Understanding intimate self-care among riverine women: qualitative research through the lens of the Sunrise Model
Source: Rev Bras Enferm. 2024 Jul 19;77(2):e20230364. doi: 10.1590/0034-7167-2023-0364 (PMC11259441; doi:10.1590/0034-7167-2023-0364)
Supplement: 0034-7167-reben-77-02-e20230364-Suppl05 [file 0034-7167-reben-77-02-e20230364-Suppl05.pdf]

TRANSCRIÇÃO DE ENTREVISTA

PRIMEIRA ENTREVISTA - GRAVAÇÃO: **P5**

1. Idade: 54 anos
2. Estado Civil: casada
3. Filhos: sim
3.1 Se sim quantos: 3
4. Escolaridade: ens. Fundamental inc.
5. Profissão: pescaria
6. Qual sua renda mensal (quantos salários-mínimos): 1 s. mínimo
7. Quantas pessoas moram na sua casa: 4

ENTREVISTA

O que você compreende quando escuta a expressão "cuidados íntimos"?

"Cuidados íntimos é higiene né... cuidar do corpo... ir ao médico" – P5

Quem lhe ensinou a ter esse tipo de cuidado?

"Minha mãe né..." – P5

E a senhora lembra idade que ela começou a falar sobre?

"é ela começou me ensinar com uns 7 anos... sobre essas coisinhas" – P5

Hum então a senhora já menstruava com 7 anos?

"Não... menstruei com 14 anos" – P5

Quais são as coisas que você faz no dia a dia que fazem parte do seu cuidado íntimo?

"tomo banho de manhã... escovo dente (pausa) meio-dia e tomo também antes de dormir" – P5

Já buscou ajuda profissional para ter mais informações sobre isso? Quais profissionais?

"Não... eu tenho vergonha um pouco de falar disso com o médico.." – P5

O que facilita ou dificulta a execução destes cuidados íntimos na sua opinião? Tipo o que pode ser difícil pra senhora fazer?

"Limpar a unha do pé (risos)" – P5

O que é inadequado na realização dos cuidados íntimos?

"hum... não sei te responder" – P5

SEGUNDA ENTREVISTA - GRAVAÇÃO: **P5**

Quais são as coisas que você faz no dia a dia que fazem parte do seu cuidado íntimo?

"Eu acordo, ai tomo banho, escovo dente... meio dia de novo tomo banho e antes de dormir tomo outro banho" – P5

O que facilita ou dificulta a execução destes cuidados íntimos na sua opinião?

"O que que facilita é que eu gosto de tomar banho né... eu pago água limpa" – P5

E o que dificulta?

"Nada" – P5

O que é inadequado na realização dos cuidados íntimos?

(Pausa)

(pergunta refeita) Depois que a senhora participou da brincadeira a senhora acha que fazia algo de inadequado"

"Não... dar pra fazer tudo certinho, o que pode ser errado é não tomar banho né" – P5
