# Supplementary material for: Understanding intimate self-care among riverine women: qualitative research through the lens of the Sunrise Model
Source: Rev Bras Enferm. 2024 Jul 19;77(2):e20230364. doi: 10.1590/0034-7167-2023-0364 (PMC11259441; doi:10.1590/0034-7167-2023-0364)
Supplement: 0034-7167-reben-77-02-e20230364-Suppl06 [file 0034-7167-reben-77-02-e20230364-Suppl06.pdf]

TRANSCRIÇÃO DE ENTREVISTA

ENTREVISTA – PÓS DINÂMICA. GRAVAÇÃO: **P6**

1. Idade: 20 anos
2. Estado Civil: solteira
3. Filhos: sim
3.1 Se sim quantos: 01
4. Escolaridade: Ensino Médio completo
5. Profissão: secretária do lar
6. Qual sua renda mensal (quantos salários-mínimos): menos de salário mínimo
7. Quantas pessoas moram na sua casa: 03 pessoas

ENTREVISTA

O que você compreende quando escuta a expressão "cuidados íntimos"?

"não sei, acho que se lavar bem..." – P6

Quem lhe ensinou a ter esse tipo de cuidado?

"minha mãe" – P6

A senhora lembra idade que começou pensar em cuidados íntimos?

"desde cedo, acho que a partir 05 anos de idade ela já me ensinava me lavar, a como eu deveria colocar o absorvente" – P6

Quais são as coisas que você faz no dia a dia que fazem parte do seu cuidado íntimo?

"eu tomo banho cedo, eu me hidrato, passo óleo no corpo, hidratante, também escovo os meus dentes." – P6

Já buscou ajuda profissional para ter mais informações sobre isso? Quais profissionais?

"não" – P6

O que facilita ou dificulta a execução destes cuidados íntimos na sua opinião?

"o que facilita é.., sei lá, aqui tem bastante água e a gente pode tomar banho toda a hora e o que dificulta lá em casa é porque eu não tenho banheiro dentro de casa..." – P6

O que é inadequado na realização dos cuidados íntimos?

"não sei, acho que não" – P6

ENTREVISTA – PÓS DINÂMICA. GRAVAÇÃO: **P6**

Quais são as coisas que você faz no dia a dia que fazem parte do seu cuidado íntimo?

“tomar banho, escovar dente, me lavar...” –P6

O que facilita ou dificulta a execução destes cuidados íntimos na sua opinião?

“ter bastante água facilita e não ter banheiro dificulta muito” – P6

O que é inadequado na realização dos cuidados íntimos?

“acho que a questão da água porque ela é muito barrenta” – P6
