# Supplementary material for: Understanding intimate self-care among riverine women: qualitative research through the lens of the Sunrise Model
Source: Rev Bras Enferm. 2024 Jul 19;77(2):e20230364. doi: 10.1590/0034-7167-2023-0364 (PMC11259441; doi:10.1590/0034-7167-2023-0364)
Supplement: 0034-7167-reben-77-02-e20230364-Suppl07 [file 0034-7167-reben-77-02-e20230364-Suppl07.pdf]

TRANSCRIÇÃO DE ENTREVISTA

PRIMEIRA ENTREVISTA - GRAVAÇÃO: **P7**

1. Idade: 42 anos
2. Estado Civil: união estável
3. Filhos: sim
3.1 Se sim quantos: 3
4. Escolaridade: ens. Fundamental inc.
5. Profissão: dona de casa
6. Qual sua renda mensal (quantos salários-mínimos): 1/2 s. mínimo
7. Quantas pessoas moram na sua casa: 4

ENTREVISTA

O que você compreende quando escuta a expressão "cuidados íntimos"?

"Cuidados íntimos acho que a pessoa tem que ter cuidado com seu corpo né... todas as regiões... quando fala intimidades fala mais da parte baixa da mulher né, se assear bem... aquela limpeza que toda mulher tem que ter" – P7

Quem lhe ensinou a ter esse tipo de cuidado?

"Minha vó que hoje é felicidade..." – P7

E a senhora lembra idade que ela começou a falar sobre?

"Olha com uns 8 a 10 anos...por aí" – P7

Quais são as coisas que você faz no dia a dia que fazem parte do seu cuidado íntimo?

"tomo banho principalmente... roupa limpa também né, tem que ter muito cuidado com nosso ambiente também de casa" – P7

Já buscou ajuda profissional para ter mais informações sobre isso? Quais profissionais?

"Olha... o profissional que a gente sempre conversa aqui é com a enfermeira (nome da profissional)" – P7

O que facilita ou dificulta a execução destes cuidados íntimos na sua opinião? Tipo o que pode ser difícil pra senhora fazer?

"Acho difícil porque a gente é muito ocupado lá com as coisas e a pescaria.... mas a gente precisa tirar um tempo pra vim aqui no posto se cuidar né" – P7

O que é inadequado na realização dos cuidados íntimos?

"Nada não" – P7

SEGUNDA ENTREVISTA - GRAVAÇÃO: **P7**

Quais são as coisas que você faz no dia a dia que fazem parte do seu cuidado íntimo?

“O banho né... limpeza com corpo, tenho cuidado com as roupas, como vocês disseram deixo pra secar as calcinhas no sol, não uso o banheiro né... hum também me cuido né, faço os exames lá da mulher... o preventivo” – P7

O que facilita ou dificulta a execução destes cuidados íntimos na sua opinião?

“O que facilita né... é ter orientação as vezes né, tipo hoje né vocês orientaram a gente tão bem, coisa que a gente nunca pensava e não sabia e hoje ficou sabendo né, tipo da calcinha secar no banheiro muita gente faz... eu sabia que não podia, mas não sabia porque agora sei que por que dar fungo né... e doenças” – P7

O que é inadequado na realização dos cuidados íntimos?

“Inadequado... é aquilo lá que vocês disseram sobre depois de ter relação sexual tem que ter o banho lá... fazer o asseio, muita gente não sabe disso, olha também foi bom saber que depois de fazer o ato sexual lá é importante fazer o xixi que vai ajudar na limpeza das partes intimas da mulher... foi muito bom a palestra... pelo menos eu né ajudou muito porque a gente não tem esse tipo de atividade com brincadeira e a gente muito agradecida de ter pessoas interessadas de vim aqui passar informações que a gente não sabia nem fazia ideia” – P7
