# Supplementary material for: Understanding intimate self-care among riverine women: qualitative research through the lens of the Sunrise Model
Source: Rev Bras Enferm. 2024 Jul 19;77(2):e20230364. doi: 10.1590/0034-7167-2023-0364 (PMC11259441; doi:10.1590/0034-7167-2023-0364)
Supplement: 0034-7167-reben-77-02-e20230364-Suppl08 [file 0034-7167-reben-77-02-e20230364-Suppl08.pdf]

TRANSCRIÇÃO DE ENTREVISTA

ENTREVISTA – PÓS DINÂMICA. GRAVAÇÃO: **P8**

1. Idade: 51 anos
2. Estado Civil: casada
3. Filhos: sim
3.1 Se sim quantos: 02
4. Escolaridade: fundamental incompleto
5. Profissão: extrativista
6. Qual sua renda mensal (quantos salários-mínimos): menos de salário mínimo
7. Quantas pessoas moram na sua casa: 03 pessoas

ENTREVISTA

O que você compreende quando escuta a expressão "cuidados íntimos"?

"tipo uma prevenção né, a gente tem que ter cuidados íntimos prevenir de doenças." – P8

Quem lhe ensinou a ter esse tipo de cuidado?

"minha mãe" – P8

A senhora lembra idade que começou pensar em cuidados íntimos?

"meus cincos anos aí ela já ensinava a gente, já ajudava a gente a fazer esses cuidados" – P8

Quais são as coisas que você faz no dia a dia que fazem parte do seu cuidado íntimo?

"eu tomo banho cedo, faço aquele asseio, aí meio dia tomo outro banho, procuro tá sempre usando loção perfume desodorante, cuidar da minha alimentação também." – P8

Já buscou ajuda profissional para ter mais informações sobre isso? Quais profissionais?

"não" – P8

O que facilita ou dificulta a execução destes cuidados íntimos na sua opinião?

"o que facilita é não ter coceira, lavar bem os meus cabelos e o que dificulta é não ter água limpa– P8

O que é inadequado na realização dos cuidados íntimos?

"não se lavar direito" – P8

ENTREVISTA – PÓS DINÂMICA. GRAVAÇÃO: **P8**

Quais são as coisas que você faz no dia a dia que fazem parte do seu cuidado íntimo?

"limpeza da região vaginal, eu aprendi que não devo usar sabonete íntimo todos os dias, agora posso usar o óleo de cocô nas relações sexuais, que não devo usar absorvente mais de quatro horas, o uso das ervas no meu asseio..." –P8

O que facilita ou dificulta a execução destes cuidados íntimos na sua opinião?

"o uso das ervas facilita os meus cuidados por que eu já tenho no mato e o que dificulta pra mim é a água" – P8

O que é inadequado na realização dos cuidados íntimos?

"A maneira de se enxugar, não colocar a toalha lá dentro da vagina" – P8
