# Supplementary material for: Understanding intimate self-care among riverine women: qualitative research through the lens of the Sunrise Model
Source: Rev Bras Enferm. 2024 Jul 19;77(2):e20230364. doi: 10.1590/0034-7167-2023-0364 (PMC11259441; doi:10.1590/0034-7167-2023-0364)
Supplement: 0034-7167-reben-77-02-e20230364-Suppl09 [file 0034-7167-reben-77-02-e20230364-Suppl09.pdf]

TRANSCRIÇÃO DE ENTREVISTA

PRIMEIRA ENTREVISTA - GRAVAÇÃO: **P9**

1. Idade: 35 anos
2. Estado Civil: casada
3. Filhos: sim
3.1 Se sim quantos: 2
4. Escolaridade: ens. Fundamental inc.
5. Profissão: garçonete
6. Qual sua renda mensal (quantos salários-mínimos): 1 s. mínimo
7. Quantas pessoas moram na sua casa: 3

ENTREVISTA

O que você compreende quando escuta a expressão "cuidados íntimos"?

"Cuidados íntimos... eu entendo que é a higiene né... tomar banho, ter cuidado durante a menstruação... é isso" – P9

Quem lhe ensinou a ter esse tipo de cuidado? E com quantos anos?

"Foi minha mãe... quando era criança... uns 6 anos" – P9

Quais são as coisas que você faz no dia a dia que fazem parte do seu cuidado íntimo?

"Tomo banho, uso o sabonete íntimo..." – P9

Já buscou ajuda profissional para ter mais informações sobre isso? Quais profissionais?

"Já o ginecologista... aqui no posto a enfermeira" – P9

O que facilita ou dificulta a execução destes cuidados íntimos na sua opinião? Tipo o que pode ser difícil pra senhora fazer?

"Dificulta... é informação né, as vezes não temos as informações do que é certo. Ah.. o que facilita esses cuidados na minha casa é ter água encanada... quando não tinha era mais dificultoso tomar banho... a agua não era bem limpa" – P9

O que é inadequado na realização dos cuidados íntimos?

"Errado... deve ser não limpar direito né" – P9

SEGUNDA ENTREVISTA - GRAVAÇÃO: **P9**

Quais são as coisas que você faz no dia a dia que fazem parte do seu cuidado íntimo?

“Tomo banho... é isso” – P9

O que facilita ou dificulta a execução destes cuidados íntimos na sua opinião?

“Olha não saber as coisas dificulta... tem as coisas que vocês falaram que eu não sabia né... tipo não poder deixar calcinha secando no banheiro, porque quando tomo banho né eu já lavava e deixava lá... tipo aquilo lá de ter que lavar as partes intimas depois das relações também... é ignorância né” – P9

E o que facilita?

“Facilita eu ter água limpa em casa... porque antes a gente se lavava com agua do rio né...” – P9

O que é inadequado na realização dos cuidados íntimos?

“Vocês disseram na palestra sobre a calcinha ficar secando no banheiro, usar lá a calça jeans muito tempo, ahh... sobre o usar sabão em barra nas partes, eu pensava que qualquer sabão podia” – P9
