# Supplementary material for: Understanding intimate self-care among riverine women: qualitative research through the lens of the Sunrise Model
Source: Rev Bras Enferm. 2024 Jul 19;77(2):e20230364. doi: 10.1590/0034-7167-2023-0364 (PMC11259441; doi:10.1590/0034-7167-2023-0364)
Supplement: 0034-7167-reben-77-02-e20230364-Suppl10 [file 0034-7167-reben-77-02-e20230364-Suppl10.pdf]

TRANSCRIÇÃO DE ENTREVISTA

ENTREVISTA – PÓS DINÂMICA. GRAVAÇÃO: **P10**

1. Idade: 17 anos
2. Estado Civil: solteira
3. Filhos: não
3.1 Se sim quantos: 0
4. Escolaridade: cursando o ensino médio
5. Profissão: estudante e secretária do lar
6. Qual sua renda mensal (quantos salários-mínimos): menos de salário mínimo
7. Quantas pessoas moram na sua casa: 04 pessoas

ENTREVISTA

O que você compreende quando escuta a expressão "cuidados íntimos"?

"há acho que se prevenir de doenças, ter cuidado pra não manter relações sem camisinha..." – P10

Quem lhe ensinou a ter esse tipo de cuidado?

"aqui no posto de saúde, a minha mãe também, a minha família..." – P10

A senhora lembra idade que começou pensar em cuidados íntimos?

"desde os nove, dez anos" – P10

Quais são as coisas que você faz no dia a dia que fazem parte do seu cuidado íntimo?

"tomar banho eu acho, limpar o rosto, cuidar da pele..." – P10

Já buscou ajuda profissional para ter mais informações sobre isso? Quais profissionais?

"não" – P10

O que facilita ou dificulta a execução destes cuidados íntimos na sua opinião?

"o que facilita mais é quando tem o material em casa e o que dificulta é quando não tem dinheiro pra comprar esses materiais– P10

O que é inadequado na realização dos cuidados íntimos?

"falta de informação" – P10

ENTREVISTA – PÓS DINÂMICA. GRAVAÇÃO: **P10**

Quais são as coisas que você faz no dia a dia que fazem parte do seu cuidado íntimo?

“deixo calcinha no varal pegando vento, aprendi que não posso mais passar o sabonete íntimo dentro da vagina e sim ao redor... –P10

O que facilita ou dificulta a execução destes cuidados íntimos na sua opinião?

“dificulta é não ter o material, tipo o sabão e facilita quando tem a água” – P10

O que é inadequado na realização dos cuidados íntimos?

“sabonete intimo em excesso, não pode deixar a calcinha no banheiro, usar muita roupa apertada na região, usar absorvente por muito tempo” – P10
