# Supplementary material for: Understanding intimate self-care among riverine women: qualitative research through the lens of the Sunrise Model
Source: Rev Bras Enferm. 2024 Jul 19;77(2):e20230364. doi: 10.1590/0034-7167-2023-0364 (PMC11259441; doi:10.1590/0034-7167-2023-0364)
Supplement: 0034-7167-reben-77-02-e20230364-Suppl11 [file 0034-7167-reben-77-02-e20230364-Suppl11.pdf]

TRANSCRIÇÃO DE ENTREVISTA

PRIMEIRA ENTREVISTA - GRAVAÇÃO: **P11**

1. Idade: 48 anos
2. Estado Civil: solteira
3. Filhos: sim
3.1 Se sim quantos: 1
4. Escolaridade: nível superior
5. Profissão: professora
6. Qual sua renda mensal (quantos salários-mínimos): 1e 1/2 s. mínimo
7. Quantas pessoas moram na sua casa: 3

ENTREVISTA

O que você compreende quando escuta a expressão "cuidados íntimos"?

"Cuidados íntimos é os cuidados com toda aquela região vagina e anus" – P11

Quem lhe ensinou a ter esse tipo de cuidado? E com quantos anos?

"Minha mãe... desde que me intendo de gente. Acho que uns 6 anos. Tanto que quando menstruei ela não ficou logo sabendo fui contar depois pra ela não brigar" – P11

Quais são as coisas que você faz no dia a dia que fazem parte do seu cuidado íntimo?

"Quando vou no banheiro fazer xixi e coco eu lavo e seco... e usar sabonete que seja próprio pra aquele cuidado" – P11

Já buscou ajuda profissional para ter mais informações sobre isso? Quais profissionais?

"Sim, quando ia na consulta ginecológica perguntava tudo, sempre fui muito curiosa e também perguntava para as amigas" – P11

O que facilita ou dificulta a execução destes cuidados íntimos na sua opinião? Tipo o que pode ser difícil pra senhora fazer?

"O que facilita é ter chuveirinho... dentro de casa tem, mas quando a gente sai nesses banheiros não tem e não pode nem se sentar. E o que dificulta é uma água de qualidade, o risco de pegar alguma coisa com a água do rio é maior" – P11

O que é inadequado na realização dos cuidados íntimos?

"Se limpar errado... muita gente não sabe a maneira certa, as vezes as mulheres não se lavam" – P11

SEGUNDA ENTREVISTA - GRAVAÇÃO:

Quais são as coisas que você faz no dia a dia que fazem parte do seu cuidado íntimo?

“Eu me lavo, principalmente depois das relações sexuais, faço meu asseio com chuveirinho depois de toda vez que faço minhas necessidades, além de usar produtos adequados pra minha limpeza” – P11

O que facilita ou dificulta a execução destes cuidados íntimos na sua opinião?

“O que facilita é ter água encanada e ter condições financeiras para comprar produtos de boa qualidade” – P11

“O que dificulta falta de saneamento básico que prejudica toda comunidade, além da falta de acesso né... de ir e vir, e o acesso a esses produtos” – P11

O que é inadequado na realização dos cuidados íntimos?

“Inadequado é a maneira de fazer a higiene, a reutilização de calcinhas sujas, o uso de absorvente por muito tempo, as vezes a mulher usa o absorvente até extravasar, o uso daquele absorvente interno que pode causar uma infecção, ah... e que vocês falaram sobre ter que fazer xixi depois da relação” – P11
