# Supplementary material for: Understanding intimate self-care among riverine women: qualitative research through the lens of the Sunrise Model
Source: Rev Bras Enferm. 2024 Jul 19;77(2):e20230364. doi: 10.1590/0034-7167-2023-0364 (PMC11259441; doi:10.1590/0034-7167-2023-0364)
Supplement: 0034-7167-reben-77-02-e20230364-Suppl12 [file 0034-7167-reben-77-02-e20230364-Suppl12.pdf]

TRANSCRIÇÃO DE ENTREVISTA

ENTREVISTA – PRÉ DINÂMICA. GRAVAÇÃO: **P12**

1. Idade: 24 anos
2. Estado Civil: solteira
3. Filhos: Sim
3.1 Se sim quantos: 01
4. Escolaridade: Cursando o ensino superior
5. Profissão: Autônoma
6. Qual sua renda mensal (quantos salários-mínimos): Menos de salário-mínimo
7. Quantas pessoas moram na sua casa: 04 pessoas

ENTREVISTA

O que você compreende quando escuta a expressão "cuidados íntimos"? A sua opinião sobre essa frase, quando a escuta.

"Que é de extrema importância a gente se cuidar, fazer sempre é... a higiene. Ter sempre a higiene cuidadinha, sempre fazer os exames de rotinas, essas coisas assim" – P12

Quem que lhe ensinou a ter esse tipo de cuidado?

"A minha mãe" – P12

A senhora lembra mais ou menos com quantos anos?

"desde bem pequena, uns 05 anos... ensinou como a gente se lava." – P12

Quais são as coisas que você faz no seu dia a dia que fazem parte do seu cuidado íntimo?

"o banho, o asseio nas calcinhas." – P12

Já buscou ajuda profissional para ter mais informações sobre isso? Quais profissionais?

"não" – P12

O que facilita ou dificulta a execução destes cuidados, para que você consiga reaiza-los?

"Não sei responder" – P12

O que você acha que pode dificultar a execução desses cuidados íntimos para que sejam feitos de maneira adequada, na sua opinião?

“Usar calcinha, por exemplo, ficar o dia inteiro sem trocar a calcinha, tipo... eu acho errado. O uso de sabonetes que não são apropriados pro nosso uso... eu acho que é só!” – P12

ENTREVISTA – PÓS DINÂMICA. GRAVAÇÃO: **P12**

Quais são as coisas que você faz no dia a dia que fazem parte dos seus cuidados íntimos?

“O asseio nas peças íntimas, calcinhas essas coisas...” –P12

O que facilita e o que dificulta a execução destes cuidados íntimos?

“não sei responder” – P12

O que é inadequado na realização dos cuidados íntimos?

“O excesso de sabão íntimo, excesso de uso de protetor de calcinha” – P12

O que a senhora achou da nossa atividade?

“Sim, foi muito importante, assim a gente aprende. Achei muito bacana!” – P12
