# Supplementary material for: Understanding intimate self-care among riverine women: qualitative research through the lens of the Sunrise Model
Source: Rev Bras Enferm. 2024 Jul 19;77(2):e20230364. doi: 10.1590/0034-7167-2023-0364 (PMC11259441; doi:10.1590/0034-7167-2023-0364)
Supplement: 0034-7167-reben-77-02-e20230364-Suppl13 [file 0034-7167-reben-77-02-e20230364-Suppl13.pdf]

TRANSCRIÇÃO DE ENTREVISTA

PRIMEIRA ENTREVISTA - GRAVAÇÃO: **P13**

1. Idade: 43 anos
2. Estado Civil: solteira
3. Filhos: sim
3.1 Se sim quantos: 2
4. Escolaridade: ens. Médico completo
5. Profissão: pescadora
6. Qual sua renda mensal (quantos salários-mínimos): 1/2 s. mínimo
7. Quantas pessoas moram na sua casa: 4

ENTREVISTA

O que você compreende quando escuta a expressão "cuidados íntimos"?

"O que acho que é cuidado íntimo é.... higienizar bem as partes, manter sempre os pelos aparadinhos" – P13

Quem lhe ensinou a ter esse tipo de cuidado?

"Minha mãe..." – P13

E com quantos anos a senhora lembra?

"Uns 7 anos" – P13

Quais são as coisas que você faz no dia a dia que fazem parte do seu cuidado íntimo?

"Olha a hora que tomo banho... meio que só isso né" – P13

Já buscou ajuda profissional para ter mais informações sobre isso? Quais profissionais?

"não" – P13

O que facilita ou dificulta a execução destes cuidados íntimos na sua opinião?

"O que dificulta é o acesso né a médico... hospital" – P13

O que é inadequado na realização dos cuidados íntimos?

"Não tem nada que acho inadequado" – P13

SEGUNDA ENTREVISTA - GRAVAÇÃO: **P13**

Quais são as coisas que você faz no dia a dia que fazem parte do seu cuidado íntimo?

"Toma banho... acho que só isso" – P13

O que facilita ou dificulta a execução destes cuidados íntimos na sua opinião?

"Facilita... não sei responder" – P13

E o que dificulta?

"O acesso que a gente não tem de informação e pra ir no médico" – P13

O que é inadequado na realização dos cuidados íntimos?

"Ah não sei..." – P13
