# Supplementary material for: Understanding intimate self-care among riverine women: qualitative research through the lens of the Sunrise Model
Source: Rev Bras Enferm. 2024 Jul 19;77(2):e20230364. doi: 10.1590/0034-7167-2023-0364 (PMC11259441; doi:10.1590/0034-7167-2023-0364)
Supplement: 0034-7167-reben-77-02-e20230364-Suppl14 [file 0034-7167-reben-77-02-e20230364-Suppl14.pdf]

TRANSCRIÇÃO DE ENTREVISTA

ENTREVISTA – PRÉ DINÂMICA. GRAVAÇÃO: **P14**

1. Idade: 32 anos
2. Estado Civil: Solteira
3. Filhos: Sim
3.1 Se sim quantos: 03
4. Escolaridade: E. médio incompleto
5. Profissão: Extrativista
6. Qual sua renda mensal (quantos salários-mínimos): Menos de salário-mínimo
7. Quantas pessoas moram na sua casa: 05 pessoas

ENTREVISTA

O que você compreende quando escuta a expressão "cuidados íntimos"?

"Compreendo como sendo uma coisa boa pra mim, o cuidado que faço comigo" – P14

Quem que lhe ensinou a ter esses cuidados?

"A minha mãe" – P14

A senhora lembra mais ou menos com quantos anos?

"eu tinha 11 anos, mais ou menos – P14

Quais são as coisas que você faz no seu dia a dia que fazem parte do seu cuidado íntimo?

"tomar um banho, lavar minhas partes íntimas, usar sabonete íntimo." – P14

Já buscou ajuda profissional para ter mais informações sobre isso? Quais eram esses profissionais?

"sim, o ginecologista" – P14

O que facilita ou dificulta a execução destes cuidados, para que você consiga realizá-los?

"facilita a gente vir aqui se tratar, ter a unidade perto, ter lugares pertos para comprar produtos. Não sei o que dificulta" – P14

O que você acha que pode dificultar a execução desses cuidados íntimos para que sejam feitos de maneira adequada, na sua opinião?

“Não sei te explicar” – P14

ENTREVISTA – PÓS DINÂMICA. GRAVAÇÃO: **P14**

Quais são as coisas que você faz no dia a dia que fazem parte dos seus cuidados íntimos?

“Quando vou no banheiro eu me lavo, tomo banho, vários no dia, passo hidratante, é isso– P14

O que facilita e o que dificulta a execução destes cuidados íntimos, na sua opinião?

“O que facilita é a gente ter acesso né ao posto de saúde, ter lugares próximos pra comprar material pra cuidar da gente. O que dificulta é a gente não ter algumas coisas, uma água encanada, é muito complicado, a água tem muita bactéria” – P14

O que é inadequado na realização dos cuidados íntimos?

“A água ser suja do rio, que faz não conseguir se limpar direito” – P14
