# Supplementary material for: Understanding intimate self-care among riverine women: qualitative research through the lens of the Sunrise Model
Source: Rev Bras Enferm. 2024 Jul 19;77(2):e20230364. doi: 10.1590/0034-7167-2023-0364 (PMC11259441; doi:10.1590/0034-7167-2023-0364)
Supplement: 0034-7167-reben-77-02-e20230364-Suppl15 [file 0034-7167-reben-77-02-e20230364-Suppl15.pdf]

TRANSCRIÇÃO DE ENTREVISTA

PRIMEIRA ENTREVISTA - GRAVAÇÃO: **P15**

1. Idade: 52 anos
2. Estado Civil: solteira
3. Filhos: sim
3.1 Se sim quantos: 3
4. Escolaridade: ens. Fundamental inc.
5. Profissão: açaí e pesca
6. Qual sua renda mensal (quantos salários-mínimos): 1/2 s. mínimo
7. Quantas pessoas moram na sua casa: 6

ENTREVISTA

O que você compreende quando escuta a expressão "cuidados íntimos"?

"Cuidados íntimos... é cuidar de mim né, eu banho" – P15

Quem lhe ensinou a ter esse tipo de cuidado? E com quantos anos?

"Olha foi minha irmã.. quando era pequena... uns 6 anos" – P15

Quais são as coisas que você faz no dia a dia que fazem parte do seu cuidado íntimo?

"Eu tomo banho" – P15

Já buscou ajuda profissional para ter mais informações sobre isso? Quais profissionais?

"Não não" – P15

O que facilita ou dificulta a execução destes cuidados íntimos na sua opinião? Tipo o que pode ser difícil pra senhora fazer?

"O dia a dia não deixa né cuidar de mim, tem que trabalhar cuidar da casa das crianças e num tenho muito tempo sabe" – P15

O que é inadequado na realização dos cuidados íntimos?

"Hum.. não sei não" – P15

SEGUNDA ENTREVISTA - GRAVAÇÃO:

Quais são as coisas que você faz no dia a dia que fazem parte do seu cuidado íntimo?

“Cuidar das calcinhas... cuidar do corpo” – P15

Teve alguma coisa que a ente falou e a senhora não sabia?

“Teve... aquela pergunta do óleo de coco... não sabia que podia usar” – P15

O que facilita ou dificulta a execução destes cuidados íntimos na sua opinião?

“O que dificulta é falta de informação né... facilita... hum não sei” – P15

“Depois que a senhora participou da brincadeira a senhora acha que fazia algo de inadequado”

“Lavar a calcinha e colocar no banheiro... eu não sabia, agora aprendi e vou colocar no sol” – P15
