# Supplementary material for: Understanding intimate self-care among riverine women: qualitative research through the lens of the Sunrise Model
Source: Rev Bras Enferm. 2024 Jul 19;77(2):e20230364. doi: 10.1590/0034-7167-2023-0364 (PMC11259441; doi:10.1590/0034-7167-2023-0364)
Supplement: 0034-7167-reben-77-02-e20230364-Suppl16 [file 0034-7167-reben-77-02-e20230364-Suppl16.pdf]

TRANSCRIÇÃO DE ENTREVISTA

ENTREVISTA – PRÉ DINÂMICA. GRAVAÇÃO: **P16**

1. Idade: 18 anos
2. Estado Civil: Solteira
3. Filhos: Não
3.1 Se sim quantos: 0
4. Escolaridade: E. médio incompleto
5. Profissão: Desempregada
6. Qual sua renda mensal (quantos salários-mínimos): Menos de um salário-mínimo
7. Quantas pessoas moram na sua casa: 02 pessoas

ENTREVISTA

O que você compreende quando escuta a expressão "cuidados íntimos"?

"ah, que a mulher tem que se cuidar mais. Não sei muito bem" – P16

Quem que lhe ensinou a ter esses cuidados?

"A minha mãe" – P16

A senhora lembra mais ou menos com quantos anos?

"depois que eu comecei a menstruar, com 12 anos – P16

Quais são as coisas que você faz no seu dia a dia que fazem parte do seu cuidado íntimo?

"tomar banho, passar sabão... acho que é isso." – P16

Já buscou ajuda profissional para ter mais informações sobre isso? Quais eram esses profissionais?

"não" – P16

O que facilita ou dificulta a execução destes cuidados, para que você consiga realizá-los?

"não sei responder" – P16

O que você acha que acha que está sendo realizado de maneira inadequada nesses cuidados?

"Tomar pouco banho, não se lavar direito... acho que só!" – P16

ENTREVISTA – PÓS DINÂMICA. GRAVAÇÃO: **P16**

Quais são as coisas que você faz no dia a dia que fazem parte dos seus cuidados íntimos?

"Tomar banho, fazer o asseio correto, lavar as calcinhas, trocar o absorvente... essas coisas" –P16

O que facilita e o que dificulta a execução destes cuidados íntimos, na sua opinião?

"Eu acho que o que facilita é ter bastante água, a gente toma vários banhos no dia, dá pra fazer o asseio. Os produtos também. O que dificulta é a água tem barro, não é limpa, e nem sempre conseguir comprar o material certo pra usar" – P16

O que é inadequado na realização dos cuidados íntimos?

"é o que foi dito né, que é usar muito tempo o absorvente, que é deixar a alcinha no banheiro, usar papel higiênico errado, o sabão íntimo, essas coisas (risos)" – P16
