# Supplementary material for: Understanding intimate self-care among riverine women: qualitative research through the lens of the Sunrise Model
Source: Rev Bras Enferm. 2024 Jul 19;77(2):e20230364. doi: 10.1590/0034-7167-2023-0364 (PMC11259441; doi:10.1590/0034-7167-2023-0364)
Supplement: 0034-7167-reben-77-02-e20230364-Suppl17 [file 0034-7167-reben-77-02-e20230364-Suppl17.pdf]

TRANSCRIÇÃO DE ENTREVISTA

PRIMEIRA ENTREVISTA - GRAVAÇÃO: **P17**

1. Idade: 43 anos
2. Estado Civil: união estável
3. Filhos: sim
3.1 Se sim quantos: 2
4. Escolaridade: ens. Médio completo
5. Profissão: garçonete
6. Qual sua renda mensal (quantos salários-mínimos): 1 e 1/2 s. mínimo
7. Quantas pessoas moram na sua casa: 3

ENTREVISTA

O que você compreende quando escuta a expressão "cuidados íntimos"?

"Cuidados é tipo o asseio né... aquele bem feito" - P17

Bem feito como?

"Ah tipo com sabonete e água limpa" - P17

Quem lhe ensinou a ter esse tipo de cuidado? E com quantos anos?

"Olha foi minha mãe... bem pequena com uns cinco anos, igual eu falo pra minha filha" - P17

Quais são as coisas que você faz no dia a dia que fazem parte do seu cuidado íntimo?

"Eu lavo bem, uso sabonete íntimos todo dia" - P17

Já buscou ajuda profissional para ter mais informações sobre isso? Quais profissionais?

"Não" - P17

O que facilita ou dificulta a execução destes cuidados íntimos na sua opinião? Tipo o que pode ser difícil pra senhora fazer?

"Eu trabalho dia todo né... não tenho tempo muito pra mim" - P17

O que é inadequado na realização dos cuidados íntimos?

"Errado é não tomar banho, não lavar direito, usar muito tempo a mesma calcinha" - P17

SEGUNDA ENTREVISTA - GRAVAÇÃO:

Quais são as coisas que você faz no dia a dia que fazem parte do seu cuidado íntimo?

“Higiene né... com o sabão (pausa)... Pois é eu tava na duvida do sabonete, eu usava né mais tinha aquele receio, agora que vocês disseram lá agora já sei” - P17

O que facilita ou dificulta a execução destes cuidados íntimos na sua opinião?

“É o tempo né que dificulta... o dia todo fora de casa, só dar de manhã e a noite” - P17

O que é inadequado na realização dos cuidados íntimos?

“Agora sei que é ficar dia todo com absorvente pra mim era normal o máximo de tempo” - P17
