# Supplementary material for: Understanding intimate self-care among riverine women: qualitative research through the lens of the Sunrise Model
Source: Rev Bras Enferm. 2024 Jul 19;77(2):e20230364. doi: 10.1590/0034-7167-2023-0364 (PMC11259441; doi:10.1590/0034-7167-2023-0364)
Supplement: 0034-7167-reben-77-02-e20230364-Suppl18 [file 0034-7167-reben-77-02-e20230364-Suppl18.pdf]

TRANSCRIÇÃO DE ENTREVISTA

ENTREVISTA – PRÉ DINÂMICA. GRAVAÇÃO: **P18**

1. Idade: 42 anos
2. Estado Civil: casada
3. Filhos: sim
3.1 Se sim quantos: 03
4. Escolaridade: nível superior completo
5. Profissão: pedagoga
6. Qual sua renda mensal (quantos salários-mínimos): dois salários-mínimos
7. Quantas pessoas moram na sua casa: 04 pessoas

ENTREVISTA

O que você compreende quando escuta a expressão "cuidados íntimos"?

"Se cuidar, né..." – P18

Quem que lhe ensinou a ter esses cuidados?

"A minha mãe" – P18

A senhora lembra mais ou menos com quantos anos?

"Desde criança, entre 05 e 06 anos, ela ensinou toda a parte de se cuidar, se lavar, por aí..." – P18

Quais são as coisas que você faz no seu dia a dia que fazem parte dos seus cuidados íntimos?

"Olha, a depilação, o banho com sabonete íntimo, a troca de calcinha, usar protetor de calcinha, a limpeza da calcinha adequadamente." – P18

Já buscou ajuda profissional para ter mais informações sobre isso? Quais eram esses profissionais?

"Não" – P18

O que facilita ou dificulta a execução destes cuidados, para que você consiga realizá-los?

"O que facilita, a questão da minha natureza, a gente se manter limpa e asseada, com meu cuidado pessoal. O que dificulta é talvez ter uma água de qualidade" – P18

O que você acha que acha que está sendo realizado de maneira inadequada nesses cuidados?

“O uso errado de banheiros público, porque as mulheres sentam, e não pode, acho que isso”– P18

ENTREVISTA – PÓS DINÂMICA. GRAVAÇÃO: **P18**

Quais são as coisas que você faz no dia a dia que fazem parte dos seus cuidados íntimos?

“Eu uso o sabonete íntimo, olho o PH, gosto muito do sabonete de coco e o glicerinado de bebê, e na minha casa só tem mulheres, usamos muito essas coisas. O asseio eu também gosto de fazer com o vinagre de maçã. Não é algo que faço diariamente, mas está dentro dos meus cuidados” –P18

O que facilita e o que dificulta a execução destes cuidados íntimos, na sua opinião?

“Eu acho que o que facilita é a necessidade né, o cuidar, o bem-estar, o cuidado pessoal. E a água de qualidade dificulta muito” – P18

O que é inadequado na realização dos cuidados íntimos?

“Vou repetir o que eu disse anteriormente, o mau uso dos banheiros público e até mesmo os da nossa própria casa, até porque algumas casas nem tem tubulação adequada. Mas também se lavar com sabonete íntimo exageradamente, limpar de forma errada com o papel higiênico, usar calcinha fio dental por muito tempo, isso mesmo]!” – P18
