# Supplementary material for: Understanding intimate self-care among riverine women: qualitative research through the lens of the Sunrise Model
Source: Rev Bras Enferm. 2024 Jul 19;77(2):e20230364. doi: 10.1590/0034-7167-2023-0364 (PMC11259441; doi:10.1590/0034-7167-2023-0364)
Supplement: 0034-7167-reben-77-02-e20230364-Suppl19 [file 0034-7167-reben-77-02-e20230364-Suppl19.pdf]

TRANSCRIÇÃO DE ENTREVISTA

PRIMEIRA ENTREVISTA - GRAVAÇÃO: **P19**

1. Idade: 27 anos
2. Estado Civil: solteira
3. Filhos: sim
3.1 Se sim quantos: 2
4. Escolaridade: ens. Médio completo
5. Profissão: rural
6. Qual sua renda mensal (quantos salários-mínimos): 1 s. mínimo
7. Quantas pessoas moram na sua casa: 2

ENTREVISTA

O que você compreende quando escuta a expressão "cuidados íntimos"?

"Acho que cuidar do meu corpo, e da higiene" – P19

Quem lhe ensinou a ter esse tipo de cuidado? E com quantos anos?

"Minha mãe... desde os 5 anos" – P19

Quais são as coisas que você faz no dia a dia que fazem parte do seu cuidado íntimo?

"Eu tomo banho e sabe... é tomo banho antes do trabalho e depois do trabalho" – P19

Já buscou ajuda profissional para ter mais informações sobre isso? Quais profissionais?

"Sim.. o ginecologista né" – P19

O que facilita ou dificulta a execução destes cuidados íntimos na sua opinião? Tipo o que pode ser difícil pra senhora fazer?

"O que facilita é quando a gente ta em casa né... tem mais tempo, mas quando ta na rua é mais difícil, e antes também quando não tinha água encanada era difícil tinha que tomar no rio ou ir pegar água no poço" – P19

O que é inadequado na realização dos cuidados íntimos?

"Acho que não usar o sabonete íntimo né... usar sabão de pedra" – P19

SEGUNDA ENTREVISTA - GRAVAÇÃO:

Quais são as coisas que você faz no dia a dia que fazem parte do seu cuidado íntimo?

“Assim como vocês disseram lá, faço a higiene correta quando vou banheiro, sabe lá da forma certa de limpar e na hora do banho” – P19

O que facilita ou dificulta a execução destes cuidados íntimos na sua opinião?

“O que dificulta é o meu tempo mesmo... mas a gente ter agua encanada facilita o banho e sabe pra não usar agua suja nas partes” – P19

O que é inadequado na realização dos cuidados íntimos?

“Hum aquilo lá do sabonete íntimo que vocês explicaram de não poder usar todo dia, eu usava todo dia, e do absorvente de ter que trocar né... as vezes por causa do tempo a gente fica dia todo com um e a calça jeans que pode dar fungo... sabe muito importante saber essas coisas pra gente não ficar fazendo” – P19
