# Supplementary material for: Understanding intimate self-care among riverine women: qualitative research through the lens of the Sunrise Model
Source: Rev Bras Enferm. 2024 Jul 19;77(2):e20230364. doi: 10.1590/0034-7167-2023-0364 (PMC11259441; doi:10.1590/0034-7167-2023-0364)
Supplement: 0034-7167-reben-77-02-e20230364-Suppl20 [file 0034-7167-reben-77-02-e20230364-Suppl20.pdf]

TRANSCRIÇÃO DE ENTREVISTA

ENTREVISTA – PRÉ DINÂMICA. GRAVAÇÃO: **P20**

1. Idade: 39 anos
2. Estado Civil: casada
3. Filhos: sim
3.1 Se sim quantos: 04
4. Escolaridade: nível fundamental incompleto (5ª série)
5. Profissão: do lar
6. Qual sua renda mensal (quantos salários-mínimos): menos de 01 salário-mínimo
7. Quantas pessoas moram na sua casa: 05 pessoas

ENTREVISTA

O que você compreende quando escuta a expressão "cuidados íntimos"?

"A pessoa se cuidar, ter mais cuidados com limpeza nas partes íntimas..." – P20

Quem que lhe ensinou a ter esses cuidados?

"foi a minha mãe" – P20

A senhora lembra mais ou menos com quantos anos?

"Eu estava com 08 anos, 09 anos, por aí." – P20

Quais são as coisas que você faz no seu dia a dia que fazem parte dos seus cuidados íntimos?

"Eu me levanto de manhã, tomo meu banho, me cuido, passo perfumes, hidratante, uso desodorante spray e tomo banho 3x no dia." – P20

Já buscou ajuda profissional para ter mais informações sobre isso? Quais eram esses profissionais?

"Não" – P20

O que facilita ou dificulta a execução destes cuidados, para que você consiga realizá-los?

"Acho assim, que a pessoa precisa ter o banho pra se manter o tempo todo limpa, com boa higiene, e a água ajuda. Acho que dificulta ela não ser muito limpa né " – P20

O que você acha que acha que está sendo realizado de maneira inadequada nesses cuidados?

“acho que a limpeza que pode estar errada. Porque as pessoas não fazem um bom tratamento, um banho adequado”– P20

ENTREVISTA – PÓS DINÂMICA. GRAVAÇÃO: **P20**

Quais são as coisas que você faz no dia a dia que fazem parte dos seus cuidados íntimos?

“Eu tomo meu banho de manhã, me lavo, lavo meu cabelo, lavo as partes íntimas, uso hidratante, desodorante, perfume, me cuido” –P20

O que facilita e o que dificulta a execução destes cuidados íntimos, na sua opinião?

“A água que a gente tem bastante né, e dá pra fazer uma boa limpeza. Acho que dificulta não ter os produtos certos pra se limpar, não ter o sabão de coco pra lavar as calcinhas, a água também que não é limpa, né, acho que isso...” – P20

O que é inadequado na realização dos cuidados íntimos?

“Acho que é a lavagem das calcinhas com sabão normal, deixar no banheiro, não trocar o absorvente direito, não se lavar direito, usar os produtos errados.” – P20
