# Supplementary material for: Understanding intimate self-care among riverine women: qualitative research through the lens of the Sunrise Model
Source: Rev Bras Enferm. 2024 Jul 19;77(2):e20230364. doi: 10.1590/0034-7167-2023-0364 (PMC11259441; doi:10.1590/0034-7167-2023-0364)
Supplement: Translation

# Translation: Compreensões sobre autocuidado íntimo com mulheres ribeirinhas: pesquisa qualitativa à luz do *Sunrise Model*

**DOI:** 10.1590/0034-7167-2023-0364pt

**Authors:** Camila Evelyn de Sousa Brito, Bruna Larissa Fernandes Coelho, Talita Lima dos Santos, Thais Cristina Flexa Souza Marcelino, Lucas Geovane dos Santos Rodrigues, Mary Elizabeth de Santana, Antonio Jorge Silva Correa, Adriana de Sá Pinheiro

**Affiliations:** I Universidade da Amazônia. Belém, Pará, Brasil; II Universidade Federal do Pará. Belém, Pará, Brasil; III Universidade de São Paulo. Ribeirão Preto, São Paulo, Brasil

**Keywords:** Atenção Primária à, Saúde, Determinantes Sociais da Saúde, Educação em Saúde, Enfermagem Transcultural, Saúde da Mulher

## Abstract

**Objetivos:**

contribuir para a mudança de compreensões e saberes do sistema popular de mulheres ribeirinhas sobre o autocuidado íntimo feminino, antes e depois da aplicação de uma dinâmica educativa.

**Métodos:**

estudo qualitativo-participativo apoiando-se no *Sunrise Model*. Participaram 20 mulheres, cadastradas em Unidade Básica de Saúde da ilha do Combu, estado do Pará, Brasil, no segundo semestre de 2022. Ocorreram entrevistas semiestruturadas antes e depois da dinâmica educativa; e posterior análise indutiva reflexiva.

**Resultados:**

aponta-se um mecanismo feminino de educação geracional; o ato cultural do banho como sinônimo de autocuidado íntimo e da prevenção de doenças; asseios íntimos com ervas medicinais; falta de abordagem do tema pelo sistema profissional; temor do uso da “água barrenta”; e falta de recursos financeiros para compra de produtos específicos voltados aos cuidados genitourinários.

**Considerações Finais:**

fatores de companheirismo e sociais movem o autocuidado íntimo, contudo tabus, desconhecimento e pobreza são vivenciados pelas ribeirinhas.

## INTRODUÇÃO

Historicamente, as populações tradicionais brasileiras que vivem às margens dos rios foram desprovidas de acesso à saúde, meios de transporte adequados, educação formal, água potável e saneamento básico, criando um ambiente desfavorável ao controle e prevenção de doenças^(1)^. Com a crise da borracha no Brasil, no final do século XIX, a mão de obra remanescente vivenciou a ausência de políticas públicas de amparo, e os trabalhadores se dispersaram nas margens dos rios. Essa migração se intensificou nas ilhas do município de Belém, onde os ribeirinhos estabelecem trocas comerciais, manejando recursos naturais e sendo referência na relação homem-natureza^(2)^.

As suas moradias são construídas com madeira (palafitas), com pouco ou nenhum acesso à energia elétrica e à água encanada, locomovem-se em barcos, canoas e “rabetas” - barcos ágeis e pequenos com motores acoplados ao casco. Ressaltam-se os riscos à saúde que sofrem pelo desfavorecimento econômico, associados aos elevados custos de transporte fluvial, tendo em vista a distância do deslocamento até municípios com mais recursos^(1)^.

Nesse sentido, destacam-se os cuidados relativos à saúde das mulheres ribeirinhas, que, além de ocuparem um papel central na subsistência do lar, precisam cuidar de si e dos familiares^(3)^. Em tal conjuntura, é importante que os enfermeiros aliem a reflexão socioantropológica às suas condutas na comunidade, reconhecendo problemas e determinantes de saúde, bem como fortificando o elo estabelecido em consultas e ações nos pontos da rede de saúde. Acredita-se que capacitações culturais promovem ou aprimoram essas competências culturais desde a formação^(4)^.

Considerando esse cenário, a higiene íntima feminina é uma forma de prevenção de doenças (corrimento vaginal, vulvovaginite, vaginose bacteriana, candidíase, tricomoníase, infecção no trato urinário, dentre outras) e de promoção à saúde, portanto é necessária a implementação de estratégias para educá-las sobre o autocuidado íntimo^(5)^. Isso posto, é pertinente abordar essa questão fundamentando se no cuidado transcultural da teoria de Madeleine Leininger (1925-2012), que amplifica a importância da prática do enfermeiro, dando-lhe mais autonomia para agir com base na complexidade do indivíduo^(6)^.

A enfermagem transcultural é desafiada a buscar por meio de seus cuidados a justiça social, considerando as temáticas do racismo, diversidade, equidade e educação em saúde^(7)^. Nessa perspectiva, o enfermeiro deve conhecer determinantes sociais em saúde para saber dimensionar e adaptar cuidados, intervenções e orientações eficazes para a comunidade. Como exemplo de populações que requerem tais condutas, têm-se os ribeirinhos, que habitam áreas originárias, às margens de rios, lagos e igarapés. Recente estudo no estado do Amazonas reforça que tais populações acessam a saúde por meio de embarcações de madeira de pequeno e médio porte; esses moradores procuram o hospital com frequência e têm dificuldades no aprazamento de consultas^(1)^.

Esta pesquisa visou trabalhar com mulheres inseridas em uma população tradicional ribeirinha, considerando seus saberes provenientes do que o sistema acadêmico denomina de “senso comum” ou “saber popular”. Para isso, foi usado o suporte conceitual do *Sunrise Model* da Teoria do Cuidado Transcultural. Madeleine Leininger apontou bases culturais do cuidar em qualquer grupalidade na promoção do bem-estar, saúde, crescimento e enfrentamento da deficiência ou morte, explicando estruturas, congruência cultural, variação intracultural e algumas universalidades^(8)^.

Sua teoria contempla a visão de mundo dos utentes (universo simbólico interno de cada grupo que qualifica formas de cuidado), dimensões da estrutura social, sistema profissional e popular. Desde o lançamento da Teoria no início da década de 1950, as pesquisas de enfermeiros lidam com modos comparativos holísticos de cuidado e cura. A teórica apela para que a enfermagem transcultural produza mais contribuições diferenciais^(8-9)^.

Diante do exposto e com a percepção empírica da deficiência no autocuidado íntimo feminino de mulheres ribeirinhas, questiona-se: Como conhecimentos seriam modificados por meio da aplicação de uma dinâmica com mulheres ribeirinhas da Ilha do Combu, no estado do Pará, para estimular a realização de autocuidados íntimos adequados?

## OBJETIVOS

Contribuir para a mudança de compreensões e saberes do sistema popular de mulheres ribeirinhas sobre o autocuidado íntimo feminino, antes e depois da aplicação de uma dinâmica educativa.

## MÉTODOS

### Aspectos éticos

A investigação seguiu a Resolução nº 510, de 7 de abril de 2016, sobre as normas de pesquisa em Ciências Humanas e Sociais, sendo aprovada pelos Comitês de Ética em Pesquisa das instituições proponentes. As participantes assinaram o Termo de Consentimento Livre e Esclarecido (TCLE); e, para preservar a confidencialidade, usou-se a codificação alfanumérica sequencial com a letra “P” de participante (P1, P2, P3 até P20).

### Referencial conceitual e metodológico

A abordagem participativa considera a experiência e determinantes locais dos participantes tendo a mediação do pesquisador. Problematizar é a chave nos encontros debate, pois o exercício participativo tem como nuance a interpretação dos participantes sobre o fenômeno. Um contexto ou enredo vinculado ao serviço e não apenas ao projeto de pesquisa é premente para permitir a devolução dos dados e análise da participação^(10)^.

Mais que uma estratégia metodológica, as rodadas, oficinas e ações que contribuam para a mudança/transformação, eclodindo experiências partilhadas, causam mediações por meio do testemunho. São uma tomada de posicionamento social do depoente e inserção da investigação em um patamar questionador. Destarte, a abordagem participativa diz que um status quo omisso interessa a uma ciência omissa, ao passo que abordagens participativas promovem estratégias de cuidado/preventivas com maior engajamento^(10)^.

A Teoria do Cuidado Transcultural tem como propósito dar explicações universais e diferenciais sobre a saúde-doença-cuidado. Sua meta é explicar ou fornecer cuidados culturalmente congruentes, seguros e significativos. Escolheu-se o *Sunrise Model* como suporte conceitual, por ilustrar os pressupostos teóricos e explicar os fundamentos do modo de cuidar intrínseco de cada grupo (valores, crenças e práticas da pessoa e das famílias)^(11)^.

A visão de mundo e as dimensões cuidativas constituem esse modelo em sete fatores: fatores tecnológicos, fatores religiosos e filosóficos, fatores de companheirismo e sociais, valores culturais e formas de vida, fatores políticos e legais, fatores econômicos e fatores educacionais. As ações de atendimento com base na apuração de tais fatores são: Preservação do cuidado cultural, Acomodação/negociação e Repadronização/reestruturação^(11)^.

### Tipo de estudo

Trata-se de pesquisa descritiva com abordagem qualitativo-participativa. Realizou-se checagem dos domínios “equipe de pesquisa e reflexividade”, “conceito do estudo” e “análise e resultados” com apoio do *Consolidated criteria for reporting qualitative research* (COREQ), validado para o Brasil^(12)^.

### Procedimentos metodológicos

#### Cenário do estudo

A geografia da Amazônia é composta por conjuntos de ilhas, dentre as quais 39 se localizam em Belém, estado do Pará (PA), Brasil. Destaca-se a Ilha do Combu, área de proteção ambiental banhada pelo Rio Guamá. O cenário foi a Unidade Básica de Saúde (UBS) Combu, que faz parte do Distrito Administrativo do Guamá (DAGUA), no furo do rio conhecido como “Igarapé do Combu”, cuja área geográfica é de aproximadamente 15 m^2(13)^. As atividades ocorrem de segunda-feira a sexta feira, das 8 h até às 16 h, com atendimento individual, dispensação de medicamentos, imunização e coleta do exame Papanicolau^(14)^.

#### Fontes de dados

A coleta de dados se deu no período de setembro a outubro de 2022. Participaram 20 mulheres, maiores de 18 anos, sexualmente ativas, que residem exclusivamente na Ilha do Combu e cadastradas na UBS.

Vale dizer que pesquisas qualitativas não devem ser pautadas apenas pela quantidade, mas também relacionadas à dimensão de fenômeno e à qualidade das ações^(15)^. A amostragem intencional não isenta o pesquisador de obter profundidade de perspectivas; e, nos procedimentos participativos, as generalizações são possíveis pela via teórico-conceitual e pelo conjunto de relações estudadas^(10)^.

Isto posto, as cinco incursões das pesquisadoras na captação da amostra ocorreram da seguinte forma: em cada incursão, quatro ou cinco mulheres cadastradas eram convidadas a responder ao roteiro antes da dinâmica; em seguida, elas participavam da estratégia educativa e depois respondiam ao roteiro após a dinâmica. Participaram da atividade 25 mulheres, porém houve uma perda de 5 mulheres, pois não tinham disponibilidade de tempo para responder aos questionamentos depois da dinâmica. Logo, por meio dessa forma de recrutamento, o estudo contou com os depoimentos de 20 mulheres, por participarem das duas etapas de coleta e ação educativa.

#### Coleta e organização dos dados

Três acadêmicas de enfermagem realizaram entrevistas semiestruturadas de nove perguntas, com duração máxima de 15 minutos. Elas estavam cientes da temática/cenário da pesquisa e foram treinadas por dois pesquisadores com experiência em pesquisas de natureza qualitativa. Destaca-se que foi o primeiro contato das pesquisadoras com as participantes. As entrevistas e a ação educativa ocorreram em sala reservada previamente, e tais etapas contaram apenas com a presença de participantes e pesquisadoras. Na primeira etapa, foram seis perguntas (pré-dinâmica) antes do jogo de tabuleiro, ocorrendo uma rodada do jogo, seguida por entrevistas com três perguntas abertas na etapa pós dinâmica. As respostas foram audiogravadas, convertidas para o formato MP3 e, posteriormente, transcritas para arquivos individuais no formato *Microsoft Word*.

#### Etapas, recursos e dinâmicas da coleta de dados

A modalidade semiestruturada segue um roteiro previamente determinado, porém adaptado ao participante^(16)^. As questões aplicadas antes da dinâmica foram: 1) O que você compreende quando escuta a expressão autocuidado íntimo feminino?; 2) Quem lhe ensinou a ter esse tipo de cuidado? Lembra da idade?; 3) Quais são as atividades que você faz no dia a dia que fazem parte do seu autocuidado íntimo?; 4) Já buscou ajuda profissional para ter mais informações? De quais profissionais?; 5) Como você executa o autocuidado íntimo feminino?; 6) O que é inadequado na realização do autocuidado íntimo feminino?. As perguntas depois da dinâmica foram: 1) Quais são as atividades que você faz no dia a dia que fazem parte do seu autocuidado íntimo?; 2) Após o jogo, como você executaria seu autocuidado íntimo?; 3) O que é inadequado na realização do seu autocuidado íntimo? Esclarece-se que a expressão “inadequado” foi usada para verificar quais foram os conhecimentos apreendidos após a dinâmica, o que pode revelar um efeito favorável da metodologia.

Os recursos pedagógicos usados incluíram: um quadro de metal feito de material magnético (Figura 1), um tabuleiro plotado composto por com 13 casas; 6 peões de imã em cores diferentes (verde, amarelo, lilás, azul, vermelho e rosa) para identificar as participantes; e um espaço destinado ao armazenamento dos cartões de perguntas. Havia ainda um dado grande (30 × 30 cm com seis lados personalizados e imagens que remetem a traços folclóricos), 24 cartões com perguntas do tipo “verdadeiro ou falso” (sobre autocuidado íntimo feminino, com as respostas no verso) e um panfleto com Regras e instruções.

**Figura 1:** Tabuleiro magnético do Caminho da Vitória-Régia, Belém, Pará, Brasil, 2023

O jogo foi desenvolvido com uma quantidade mínima de dois e máxima de seis participantes, devendo ser concluído em até 20 minutos. As artes dos materiais, desenvolvidas na plataforma Canva Pro, têm como base jogos de tabuleiro. Os cartões são numerados e com imagens no verso (12 assertivas adequadas e 12 assertivas inadequadas), baseadas em literaturas relacionadas ao autocuidado íntimo feminino. Os eixos escolhidos foram: anatomia genital feminina, higiene genital, tipos de corrimento (e o que ocasiona), utilização de preservativos, infecções sexualmente transmissíveis e exame Papanicolau. O nome “Caminho da Vitória-Régia” foi devido à flor Vitória régia ser proveniente da região Amazônica, uma planta aquática com influência no folclore da Região Norte do Brasil, simbolizando a devoção da indígena Naiá à deusa Lua, Jacy^(17)^.

#### Análise de dados

Neste estudo, não se empregaram softwares: a análise foi manual com validação de quatro codificadores, emergindo do conjunto de depoimentos. Utilizou-se o referencial indutivo de conteúdo de seis etapas e do tipo análise temática reflexiva^(18-19)^. Assim, em conformidade com o *Sunrise Model* e com a estratégia participativa, os dados do momento anterior e do momento posterior à dinâmica foram analisados separadamente, para verificar quais foram as modificações de saberes. Familiarizou se com as transcrições em busca de padrões na leitura de cada entrevista transcrita individualmente em *Microsoft Word*; e, posteriormente, uniram-se as entrevistas individuais em um arquivo *Microsoft Word* único (matriz); geraram-se códigos com base na identificação de “códigos latentes” (aqueles além do significado semântico dado pelos participantes, que fornece uma interpretação sobre o conteúdo) desvelando os sete fatores de Leininger com uma linha de raciocínio indutivo; buscaram-se temas, nos quais os códigos gerados na fase anterior foram agrupados por proximidade de significados e segregados em outros arquivos *Microsoft Word*; ocorreram refinamentos e conferências dos temas gerados com releitura dos arquivos; houve a definição e atribuição de nomes aos temas considerando a transculturalidade; e obteve-se o relatório final. Tais etapas ocorreram no arquivo matriz dos depoimentos anteriores e posteriores à dinâmica.

## RESULTADOS

Das 20 mulheres, a maioria era solteira, com faixa etária predominante entre 25 e 45 anos, com uma média de dois filhos. Sobrevivia com uma renda mensal de 300 a 600 reais (menos de um salário mínimo atual, que é de 1.302 reais), sendo a pesca e o extrativismo as profissões predominantes, com aumento de renda na época da colheita do açaí (*Euterpe oleracea*).

A análise dos dados culminou na construção do Quadro 1. Explicando os temas criados à luz do conhecimento científico de Leininger e da coleta em duas etapas, identificam-se os seguintes fatores de cuidado^(8)^: Tema 1 - fatores de companheirismo e sociais, valores culturais e modos de vida, fatores educacionais e fatores tecnológicos; Tema 2 - fatores religiosos e filosóficos, fatores políticos e legais e fatores econômicos. Os códigos latentes relacionados à preservação, acomodação e repadronização foram agrupados no Tema 3, contemplando as decisões e ações de cuidado transcultural do *Sunrise Model*.

**Quadro 1:** Temas desvelados antes e depois da estratégia educativa, Belém, Pará, Brasil, 2023

Momento de produção dos dados	Temas
Depoimentos pré-dinâmica	1) Associação entre cultura e educação nas formas tradicionais de obtenção do conhecimento
	2) O impacto de convicções e de recursos externos favoráveis e desfavoráveis para os cuidados íntimos
Depoimentos pós-dinâmica	3) Verificação da necessidade de ajustes, manutenção e ensino de outros meios de cuidados femininos congruentes usando o Caminho da Vitória-Régia

### Pré-dinâmica: Associação entre cultura e educação nas formas tradicionais de obtenção do conhecimento

Reportaram-se fatores de companheirismo e sociais quando se recordaram de que suas mães e avós as educaram. São ensinamentos enraizados nos vínculos familiares femininos.

*Minha mãe me ensinou quando eu comecei a menstruar.* (Síntese P1, P2, P3, P4, P8)

*Minha mãe, acho que a partir dos 5 anos de idade, ela já me ensinava a me lavar, igual eu ensino minha filha agora.* (Síntese P3, P6, P17)

*Minha vó, que hoje é falecida, me ensinou com uns 8 a 10 anos.* (Síntese P7, P14, P16)

*Foi minha mãe quando eu era criança. Acho que eu tinha por volta de uns 6 anos.* (Síntese P9, P10, P11, P13, P15, P18, P20)

*Quem me ensinou foi minha irmã mais velha.* (Síntese P12, P19)

Apesar dos ensinamentos do sistema popular, sentem necessidade de palestras e informações profissionais, visto que manifestam indecisão na hora da escolha de produtos de higiene.

*O que facilita os cuidados íntimos é o acesso às informações corretas, tipo palestra.* (Síntese P1, P2, P10)

*Sobre ter muitos sabonetes íntimos* [...] *assim, né* [...] *a pessoa fica indecisa por qual usar, porque, tipo, tem uns que podem dar alergia e tem uns que podem fazer coçar, e isso me incomoda bastante, e os absorventes também.* (Síntese P3, P12)

Como fatores educacionais, destacaram-se o autocuidado íntimo feminino relacionado apenas à higiene pessoal, sendo o banho o principal meio de execução. O “cuidado na menstruação” alude ao que é considerado incorreto, como o mal asseio da região genital.

*Fazer a limpeza errada* [...] *muita gente não sabe a maneira certa; às vezes, as mulheres não se lavam e não sabem muito bem como que devem fazer.* (Síntese P9 e P11)

*O uso errado de banheiros públicos, porque as mulheres se sentam, mas não pode; acho que não se lavar depois de ir ao banheiro também está errado* [...] *vai que pega doença.* (P18)

*Tenho que fazer uma boa higiene, né, tomar banho, ter cuidado na menstruação, lavar as partes íntimas, porque tenho que ir ao médico me consultar, fazer a minha depilação.* (Síntese P1, P2, P4, P5, P9, P13, P17, P20)

Algumas relacionaram autocuidado íntimo feminino com a prevenção de infecções sexualmente transmissíveis e algumas doenças do sistema genital, porém não identificaram ou nomearam quais.

*Acho que se prevenir de doenças* [...]. *Ter cuidado para não manter relações sem camisinha e acabar se contaminando.* (Síntese P8 e P10)

*Acho difícil ter tempo para me cuidar porque a gente é muito ocupado lá com as coisas e também na pescaria* [...] *mas a gente precisa tirar um tempo pra vir aqui no posto.* (P7)

Conforme fatores culturais e modo de vida em concordância com fatores educacionais, o banho é o principal e, às vezes, único ato de cuidados íntimos que elas realizam:

*Faço meus cuidados íntimos no banho* [...]. *Tipo de manhã, quando eu levanto, eu faço a minha higiene: passo um sabonete íntimo e depois troco de roupa; mais tarde, tomo um banho e torno a me lavar, tomando vários banhos, mas não uso sabonete íntimo em todos os banhos* [...] *também troco o meu absorvente de duas em duas horas quando estou menstruada.* (Síntese P1, P3, P4, P5, P7, P8, P9, P12, P14, P15, P16, P17, P18, P19, P20)

*Quando vou no banheiro fazer xixi e cocô, eu lavo e seco as minhas partes; e também tem que usar sabonete que seja próprio para aquele cuidado.* (P11)

Dentre os fatores tecnológicos na busca de cuidados e orientações, percebe-se a relutância da maioria em procurar o sistema profissional para conversar sobre o tema.

*Já eu, sempre me consulto com o médico ginecologista. Quando é aqui no posto, procuro a enfermeira!* (Síntese P1, P2, P9, P11, P19)

*Nunca procurei informações sobre essas coisas, nem com médicos e nem com pessoal de hospital.* (Síntese P3, P4, P5, P6 P7, P8, P10, P12, P13, P14, P15, P16, P17, P18, P20)

### Pré-dinâmica: O impacto de convicções e de recursos externos favoráveis e desfavoráveis para os cuidados íntimos

Três das participantes sentem constrangimento ao conversar sobre o tema com profissionais homens; assim, assuntos como menstruação e sexualidade são tabus.

*Eu acho inadequado. É que eu não gosto muito é de conversar sobre essas coisas, principalmente da parte intima, com homem médico* [...]. *Vergonha.* (Síntese P1, P5 e P12)

Os fatores políticos e legais frequentemente relacionados aos fatores econômicos são mencionados na falta de infraestrutura adequada em lugares públicos ou trabalho do lar, como dificultadores ou facilitadores dos cuidados íntimos.

*O que facilita é não ter coceira, não ter infecção, lavar bem minhas partes; quando tem o material em casa, às vezes não temos as informações do que é certo, ir no médico, no hospital. O que dificulta é quando não tem dinheiro; outra coisa que facilita esses cuidados é ter água encanada. Quando não tinha, era mais dificultoso, porque a água não era bem limpa. Quando não tinha água encanada, era difícil; tinha que tomar no rio ou ir pegar água no poço.* (Síntese P4, P8, P9, P10, P13 e P19)

*O que facilita é que aqui tem bastante água, e a gente pode tomar banho toda hora; e o que dificulta lá em casa é porque eu não tenho banheiro dentro de casa.* (P6)

*Tem que trabalhar, cuidar da casa, das crianças, e não tenho muito tempo pra isso. Eu trabalho o dia todo.* (Síntese P15 e P17)

*O que facilita é ter chuveirinho* [...] *dentro de casa, tem. E o que dificulta é não ter uma água de qualidade: o risco de pegar alguma coisa com a água do rio é maior.* (P11)

### Pós-dinâmica: Verificação da necessidade de ajustes, manutenção e ensino de outros meios de cuidados femininos congruentes usando o Caminho da Vitória-Régia

As mulheres, após a atividade proposta, referiram uso de ervas típicas da região para o auxílio na limpeza do corpo, relatando a facilidade no acesso e os benefícios decorrentes da utilização.

*Tomo banho, me asseio, faço com camomila* [*Matricaria recutita*]*, alecrim* [*Salvia rosmarinus*] [...] *é isso que facilita, porque é fácil de conseguir.* (P1)

*O uso das ervas facilita os meus cuidados porque eu já tenho no mato; eu planto hortelã* [*Mentha spicata*]*, alecrim, jambú* [*Acmella oleracea*]. (P8)

*Uso umas ervas que tem no quintal; às vezes, faço o banho, sabe, tipo alecrim, alfavaca [Ocimum basilicum], erva-doce [Pimpinella anisum]. As vizinhas também têm. O que é ruim mesmo era quando não tinha a água limpa.* (P13)

Mesmo após a atividade, as participantes acreditaram que o autocuidado íntimo feminino se resume exclusivamente à prática do banho e que um dos facilitadores é a água encanada nas casas.

*Eu acordo, aí tomo banho, escovo dente* [...]. *Quando vou no banheiro, eu me lavo, tomo banho, várias vezes ao dia; passo hidratante, mantenho a limpeza, a higiene; meio-dia, de novo tomo banho e, antes de dormir, tomo outro banho. O que facilita é que eu gosto de tomar banho, né* [...] *eu pago água limpa.* (P3, P5, P6, P14, P19, P20)

*O que facilita é ter água encanada e ter condições financeiras para comprar produtos de boa qualidade, já o que dificulta é a falta de saneamento básico que prejudica toda a comunidade - antes a gente se lavava com água do rio, né.* (P9, P11)

*O banho, né* [...] *limpeza do corpo, tenho cuidado com as roupas, como vocês disseram, lavo minhas calcinhas, deixo pra secar as calcinhas no sol, troco absorvente, não uso o banheiro público, né* [...]. *Faço os exames lá da mulher* [...] *o preventivo.* (P7, P12, P15, P16)

*Eu me lavo, principalmente depois das relações sexuais; faço meu asseio com chuveirinho depois de toda vez que faço minhas necessidades.* (P11)

Ajuste do autocuidado íntimo feminino adequado foi relacionado ao uso correto de produtos de higiene e ao manejo de técnicas e materiais para cuidado íntimo e corporal. Esse resultado foi obtido após a aplicação da dinâmica educativa.

*O que vocês falaram do sabonete íntimo* [...] *que é comum a gente ver sabonete íntimo e pensa que a gente pode tá utilizando direto, e acaba que não, que já tinha ouvido falar do PH, mas eu pensei que era o sabonete em barra que alterava. Agora não, eu fiquei sabendo que o sabonete íntimo também altera* [...]. *Eu não imaginaria isso do sabonete íntimo.* (P1)

*Ter água limpa e os produtos certos facilita a limpeza da região vaginal. Eu aprendi que não devo usar sabonete íntimo todos os dias nem dentro da vagina, só nos arredores, que agora posso usar o óleo de coco nas relações sexuais, que eu não devo usar absorvente mais de quatro horas nem protetor de calcinha todos os dias nem calcinha fio dental. O uso das ervas no meu asseio facilita, assim como a depilação, deixar a calcinha secando no varal, e o que dificulta é não ter um banheiro, não ter dinheiro pra comprar os materiais.* (Síntese P4, P6, P8, P10, P12, P13, P16, P17, P18, P20)

*Inadequado é a reutilização de calcinhas sujas, o uso de absorvente por muito tempo. Às vezes, a mulher usa o absorvente até extravasar, o uso daquele absorvente interno que pode causar uma infecção; vocês falaram sobre ter que fazer xixi depois da relação.* (P11)

*O que facilita é a gente ter acesso ao posto de saúde, ter lugares próximos para comprar material. O que dificulta é a gente não ter água encanada. Agora eu sei que a água tem muita bactéria, e a gente pode pegar alguma doença.* (P14)

A repadronização de conhecimentos diante da falta de conhecimento do autocuidado íntimo feminino foi amplamente relatada.

*O que facilita é ter orientação, tipo hoje: vocês orientaram a gente tão bem, coisa que a gente nunca pensava e não sabia e hoje ficou sabendo, tipo da calcinha secar no banheiro muita gente faz* [...] *eu sabia que não podia, mas não sabia porque, agora eu sei, que é porque pode dar fungo e doenças. Aprendi sobre os sabonetes certos, o absorvente, o preventivo que precisa fazer. O óleo de coco que aprendi que posso usar, não enxugar dentro da vagina.* (Síntese P3, P7, P8, P9, P10, P13, P15, P16, P17, P19, P20)

*Inadequado é aquilo que vocês ensinaram, sobre depois de ter relação sexual tem que ter o banho, fazer o asseio. Muita gente não sabe disso! Olha, também foi bom saber que depois de fazer o ato sexual é importante fazer o xixi, que vai ajudar na limpeza das partes íntimas da mulher.* (P7)

## DISCUSSÃO

Sob uma perspectiva histórica, os conceitos do Brasil Colônia e República fundamentam a compreensão da Amazônia e seus povos. Atualmente, movimentos nacionais e internacionais influenciam mudanças no desenvolvimento educacional dessas populações, de modo que os saberes baseados exclusivamente no seio da cultura indígena são mesclados aos traços culturais diversos^(20)^. A estrutura familiar tradicional acomodou o homem como principal provedor, contudo esse quadro foi alterado no Ocidente mediante o trabalho feminino fora de casa. No contexto ribeirinho, a mulher ajuda diretamente no produto econômico familiar, protagonizando a educação de outras mulheres como fator de companheirismo, valores culturais e modos de vida. Estas aprenderam sobre seu corpo com suas mães e avós, por haver um dever implícito de transmitir tais cuidados e ensinamentos^(21)^.

Esse traço é bem marcante e mostra-se diverso na cultura ribeirinha, se comparado ao caso de jovens mulheres filipinas, em sua maioria católicas, vivendo nos Estados Unidos: elas reportaram que o aprendizado sozinhas ou mesmo com suas mães era reticente, prático, pouco explicativo, possivelmente em razão da cultura “tradicional” asiática^(22)^. Estudo em realidade indiana constatou, semelhantemente, que as jovens têm pouca abertura para falar de menstruação com a família e mesmo com ginecologistas, corroborando que, quando meninas e adolescentes são mal orientadas ou não dialogam sobre o tema, geralmente desenvolvem distúrbios genitourinários^(23)^.

Sob os aspectos educacionais do *Sunrise Model*, infere-se que as problemáticas existentes necessitam da implantação de políticas microssocias adequadas. O Ministério do Desenvolvimento Social (MDS) brasileiro instituiu a Política Nacional de Desenvolvimento Sustentável dos Povos e Comunidades Tradicionais (PNPCT) por meio da Comissão Nacional de Desenvolvimento Sustentável das Comunidades Tradicionais (CNPCT), mediante o Decreto 6.040, de 7 de fevereiro de 2017^(24)^.

O modo pelo qual as mulheres interpretam a materialidade dos cuidados difere entre si, mas parte da mesma perspectiva paradigmática, na qual símbolos culturais e naturais se integram no agir, no ato cultural do “banho” definido pelo rio. O SUS, para diminuir a vulnerabilidade, se propõe a valorizar práticas e conhecimentos tradicionais rurais-urbanos^(11,25)^. Neste estudo, a prevenção de doenças parte de uma cosmologia que não sabe e não nomeia exatamente aquilo que o sistema acadêmico-biomédico fixou: infecção urinária, vaginites, infecção sexualmente transmissível^(26)^.

Além disso, três mulheres da amostra mencionaram constrangimento em conversar com profissionais do sexo masculino. Existem consequências da criação patriarcal rural: a vergonha de seus genitais e, algo mais profundo, a violação da intimidade. Semelhantemente, o fator patriarcal persiste em culturas asiáticas; e o gênero feminino ter (ou não) confidencialidade é um fator fundamental para a procura de um profissional^(22)^. A vergonha pode ser combatida em grupos de mulheres, tornando mais fácil que se submetam a procedimentos invasivos como o rastreio no Papanicolau; e pode haver o encorajamento com ações e conversas em roda para desmitificações^(27)^.

Nos verbatins do segundo tema, relatou-se a aspiração à agua limpa e o fato de a água barrenta ser prejudicial. Essa necessidade encontra amparo no Art. 2 da PNPCT, que dispõe sobre o objetivo principal de promover o desenvolvimento sustentável, com ênfase na garantia de direitos. A implementação da política impulsionou o MDS na aplicação de medidas diante dos principais problemas das comunidades tradicionais, como o apoio a projetos que auxiliam a estruturação da produção familiar, a comercialização e a ampliação do acesso a água^(24)^. O deslocamento por rios e as atividades de subsistências desenvolvidas fazem com que o tempo disponível seja um obstáculo na busca do sistema formal^(1)^. A participação social dessas mulheres as estabelece como categoria específica na luta contra a exclusão física e simbólica, por estarem ligadas ao rio. No Pará, essa participação social tem se fortalecido devido ao Programa Nacional de Apoio à Agricultura Familiar (PRONAF), Movimento Paraense de Educação no Campo e Fórum Paraense de Educação no Campo.

Fatores econômicos são impeditivos para a compra de produtos, relegando as mulheres ao constrangimento e à “pobreza menstrual”. O termo refere-se a milhões de pessoas menstruantes que, devido ao perfil socioeconômico não podem arcar com produtos de higiene, não possuem água potável ou mesmo saneamento que possibilite asseio íntimo digno, fazendo com que esse processo natural seja penoso e cause, inclusive, afastamento do trabalho ou escola durante o ciclo^(28-29)^. Uma via encontrada no saber popular são os banhos com ervas, originários da tradição indígena, privilegiando uma terapêutica natural que se baseia na territorialidade, religiosidade e rituais de cura, o que compõe a cosmologia ribeirinha^(25)^. O asseio íntimo com ervas enraíza-se em um modo de produzir saúde geracional e milenar; e, mesmo com avanços farmacológicos, é uma utilização corriqueira^(30)^.

Isso se assemelha a um estudo realizado em Gana, país africano, no qual se mostrou que mulheres possuem percepções positivas sobre ervas medicinais partindo dos conhecimentos indígenas, dos vizinhos, família e da própria autonomia. O uso de ervas deve-se ao custo-efetividade e à disponibilidade relativos às seguintes plantas: camomila (*Matricaria recutita*), alecrim (*Salvia rosmarinus*), hortelã (*Mentha spicata*), jambú (*Acmella oleracea*), alfavaca (*Ocimum basilicum*) e erva-doce (*Pimpinella anisum*)^(31)^. Nesse ponto, na ótica do *Sunrise Model*, há uma confluência entre os fatores econômicos e os valores culturais e modos de vida.

Outra pesquisa^(32)^ defende a utilização de ervas medicinais, frisando que esses saberes estão consideravelmente enraizados na cultura e que, mesmo tendo acesso a medicamentos modernos, a opção pela utilização das ervas é resultado de uma escolha pessoal. Assim, percebe-se que as ervas camomila, hortelã, erva-doce, alecrim, alfavaca, mencionadas pelas participantes, estão presentes nos artigos citados anteriormente, indicando a proximidade cultural entre esses contextos quando se trata do tratamento de doenças.

Semelhantemente, comparando os resultados com a aplicação do *Sunrise Model* em vilas espanholas, foi dominante o tema “medidas de higiene”, desvelando que as mulheres lançam mão dos recursos animais, vegetais e minerais para a limpeza dos corpos; e o tema “companheirismo”, mostrando como a comunidade tem o potencial de acolher tais práticas cuidativas. Por fim, acredita se que os dados reforçam a necessidade de ações de enfermagem voltadas ao autocuidado íntimo feminino geracional, nem sempre visibilizado pelo sistema acadêmico, destacando-se, no sistema popular as mulheres mais velhas como transmissoras desse saber.

### Limitações do estudo

As entrevistas estiveram condicionadas à espera pelas consultas e dinâmica do atendimento na UBS, logo os dados produzidos no contexto da atividade foram significativamente afetados por isso. Possivelmente, o maior tempo de disponibilidade para depoimentos e a aplicação da dinâmica em um cenário diferente favoreceriam outras imersões temáticas.

### Contribuições para a Área

A teoria transcultural contribui para a identificação de problemas de enfermagem, convertendo o cuidado cultural em um princípio norteador do Processo de Enfermagem^(33-34)^. Ao aplicar o jogo Caminho da Vitória-Régia, ficaram explícitas as lacunas de conhecimento formal sobre o autocuidado íntimo feminino. Salienta-se a figura do enfermeiro como principal mediador no compartilhamento de informações dentro da Atenção Primária à Saúde (APS), sendo que a pesquisa ajuda na identificação das reais necessidades, pautadas nas limitações locais. Compreendeu-se que é preciso, nas práticas em saúde, a congruência do cuidado cultural e o cumprimento dos princípios da alteridade, sabendo que a APS deve preservar a maioria das compreensões. A educação em saúde é uma importante arbitragem para conscientização desses povos da água e da mata, que não ignoram a necessidade de ensinamentos do sistema técnico-científico e atestam a relevância do conhecimento popular recebido dos seus semelhantes, mesmo sabendo que não são suficientes no que tange ao autocuidado íntimo feminino.

## CONSIDERAÇÕES FINAIS

Foi modificado o conhecimento de mulheres ribeirinhas sobre o autocuidado íntimo feminino, entretanto não se buscou excluir as suas vivências e costumes, que, em uma análise mais profunda, precisam ser reconhecidos como patrimônio cultural a ser preservado, ratificando as políticas públicas do SUS na área do multiculturalismo. Chama a atenção nesses saberes a vocalização de como a educação participativa facilitou a compreensão das informações que são, segundo fatores de companheirismo e sociais, apenas repassadas por mães, irmãs, tias e avós em algum momento da infância-puberdade, sendo as mães as principais transmissoras desses saberes geracionais e quase instintivos sobre o corpo. O Caminho da Vitória-Régia visibilizou em um momento-espaço o “sistema acadêmico-profissional” junto com o “sistema popular-usuários”, uma forma de produção de saúde.

Os fatores econômicos foram fortemente elencados pelas participantes, citando a falta de acesso à água encanada como um dos mais relevantes no momento da prática de seus autocuidados íntimos femininos. Logo, durante a realização da sua higiene como repadronização do cuidado cultural no âmbito dos conhecimentos, relatou-se a presença de certo temor por utilizarem na limpeza da vulva a “água barrenta” do rio, já que esta é não tratada e contaminada. Assim, revelam que estão cientes de que não é adequada por conter microrganismos que favorecem infecções.

Segundo *Sunrise Model* de Madeleine Leininger, as participantes conseguiram receber novos conhecimentos e, certamente, aplicarão e difundirão no seu convívio social-geracional as formas adequadas de autocuidado íntimo feminino, ainda em sinergia com a natureza que cerca a ilha do Combu.

## Data Availability

https://doi.org/10.48331/scielodata.8D4CF7
